# Split Histidine Kinases Enable Ultrasensitivity and Bistability in Two-Component Signaling Networks

**DOI:** 10.1371/journal.pcbi.1002949

**Published:** 2013-03-07

**Authors:** Munia Amin, Steven L. Porter, Orkun S. Soyer

**Affiliations:** 1 Biosciences, College of Life and Environmental Sciences, University of Exeter, Exeter, United Kingdom; 2 Systems Biology Program, College of Engineering, Computing and Mathematics, University of Exeter, Exeter, United Kingdom; North Carolina State University, United States of America

## Abstract

Bacteria sense and respond to their environment through signaling cascades generally referred to as two-component signaling networks. These networks comprise histidine kinases and their cognate response regulators. Histidine kinases have a number of biochemical activities: ATP binding, autophosphorylation, the ability to act as a phosphodonor for their response regulators, and in many cases the ability to catalyze the hydrolytic dephosphorylation of their response regulator. Here, we explore the functional role of “split kinases” where the ATP binding and phosphotransfer activities of a conventional histidine kinase are split onto two distinct proteins that form a complex. We find that this unusual configuration can enable ultrasensitivity and bistability in the signal-response relationship of the resulting system. These dynamics are displayed under a wide parameter range but only when specific biochemical requirements are met. We experimentally show that one of these requirements, namely segregation of the phosphatase activity predominantly onto the free form of one of the proteins making up the split kinase, is met in Rhodobacter sphaeroides. These findings indicate split kinases as a bacterial alternative for enabling ultrasensitivity and bistability in signaling networks. Genomic analyses reveal that up 1.7% of all identified histidine kinases have the potential to be split and bifunctional.

## Author Summary

Two-component signaling systems mediate many of the physiological responses of bacteria. In their core, these systems consist of a histidine kinase (HK) and a response regulator (RR) that it can phosphotransfer to. Around this core interaction, evolution has led to several conserved biochemical and structural features. In order to achieve a broad and predictive understanding of bacterial signaling, it is important to assess whether these features enable specific signaling dynamics and properties. Our study provides a potential functional role for one such feature, the split histidine kinases, where autophosphorylation and phosphotransfer activities of a conventional HK are segregated onto distinct proteins capable of complex formation. We show that that this unusual configuration can enable ultrasensitivity and bistability in signal transduction under specific biochemical conditions. We experimentally show that one of these requirements, namely segregation of the phosphatase activity predominantly onto the free form of one of the proteins making up the split kinase, is met in proteins isolated from *Rhodobacter sphaeroides*. Genomic studies suggest 1.7% of all histidine kinases could function as bifunctional split kinases. This study provides a linkage between these proteins and response dynamics, thereby enabling experimentally testable hypotheses in these systems.

## Introduction

Bacterial responses to many external stimuli are underpinned by two-component signaling networks (TCSNs). These are found in most bacterial species and are also present in Archaea, eukaryotic microbes, and plants [1], [2]. TCSNs are built upon the core reactions involving a histidine kinase (HK) that autophosphorylates on a conserved histidine residue in response to a signal, and a cognate response regulator (RR) that is activated when the HK phosphorylates one of its conserved aspartate residues [3]. Evolutionary processes seem to have exploited the modular structure of these proteins to produce a distinct set of biochemical features and network structures that reoccur in diverse TCSNs; bifunctional HKs [4], sink RRs [5], phosphorelays [6] and split HKs [7]. In order to achieve a broad and predictive understanding of bacterial signaling, it is important to assess whether these features enable specific signaling dynamics and properties [8].

There has already been progress towards this goal. Firstly, bifunctional HKs, which display both phosphatase and kinase activity towards their cognate RR, enable robustness in system output with respect to fluctuations in the amount of these signaling proteins [4], [9] and reduce cross-talk among different TCSNs [10], [11]. Further, theoretical work indicates that bi-functional HKs can generate flexible signal-response relationships [12], [13] and allow higher signal amplification compared to monofunctional HKs that lack phosphatase activity [10]. Secondly, sink RRs, which compete with another RR for phosphoryl groups from a single cognate HK, are suggested to allow faster response termination [5], [14]. Finally, phosphorelays, which contain several proteins (or domains) acting as a relay between the HK and RR, are suggested to integrate several signals received on their different layers [15]–[17] and implement both ultrasensitive and linear responses [18], [19]. Taken together, these studies suggest that specific biochemical and structural features in TCSNs could enable specific functional roles.

**Figure 1:**
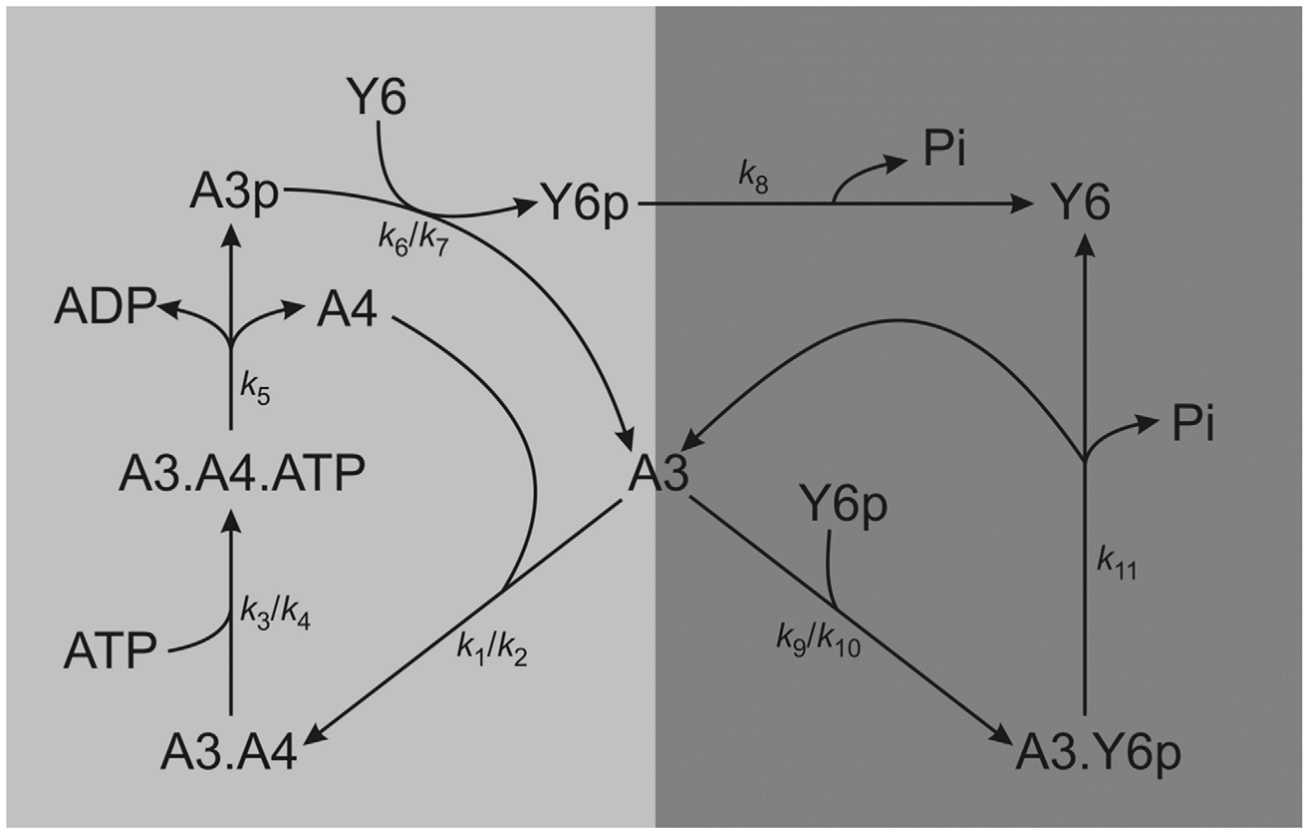
A cartoon diagram of the CheA3-CheA4-CheY6 split kinase system. The diagram is arranged so to highlight the role of free CheA3 acting as a branching point for the two arms that form competing cycles leading to phosphorylation and dephosphorylation of CheY6. Rate constants are shown on the relevant reactions. In the case of reversible reactions, two rate constants are given (*k*_forward_/*k*_reverse_).

Of the different features of TCSNs, split kinases are predicted in several bacterial genomes [1], [2] and are biochemically characterized in *Rhodobacter sphaeroides* [7], [20]. In this organism, the split kinase system is composed of CheA3 and CheA4, which form a bipartite histidine kinase that phosphorylates the response regulator CheY6 [21] (Figure 1). CheA4 lacks the phosphorylatable P1 domain, whereas CheA3 lacks the dimerization (P3) and catalytic kinase (P4) domains. Neither CheA3 nor CheA4 can autophosphorylate when incubated separately with ATP; however, when a mixture of CheA3 and CheA4 is incubated with ATP, then CheA3 becomes phosphorylated, indicating that these proteins can act as a histidine kinase only by forming a complex [21]. Activated by incoming signals, the P4 domain of CheA4 binds ATP and phosphorylates the P1 domain of CheA3. Subsequently, CheA3-P acts as a phosphodonor for its cognate response regulator, CheY6 [21], which controls flagellar rotation [22]. In vivo, CheA3 and CheA4 co-localize to the cytoplasmic chemotaxis cluster [23] and are both essential for chemotaxis [7], [24]. CheA3 and CheA4 bind to the cytoplasmic cluster via their P5 domains [25]. Whilst part of this cluster, CheA3 and CheA4 dynamically interact with one another. To allow phosphorylation of CheA3, the P4 domain of CheA4 must transiently bind to the P1 domain of CheA3 (in the subsequent analysis we refer to this complex as CheA3:CheA4). Once phosphorylated, the P1 domain of CheA3 is released by CheA4, and CheA3-P can then donate its phosphoryl group to the corresponding response regulator CheY6 [21], [26]. In addition to its phosphotransfer function, CheA3 is also a phosphatase towards CheY6-P [7]. *cheA3* mutants retaining phosphotransfer functions but lacking phosphatase activity do not support chemotaxis, similarly, *cheA3* mutants retaining phosphatase activity but lacking phosphotransfer activity also fail to support chemotaxis, indicating that chemotaxis requires both activities of CheA3 [7], [21]. In addition, to being phosphorylated and dephosphorylated by the split kinase comprising CheA3 and CheA4 [21], CheY6 is also phosphorylated by CheA2 at the polar chemotaxis cluster [27].

Despite this wealth of information, the general role of split kinases in bacterial signaling is not clear. In essence split kinases are unusual bifunctional HKs, where the autophosphorylation and subsequent phosphotransfer and phosphatase activities are encoded on two separate proteins. Since the complex formed by these proteins is functionally equivalent to a bifunctional HK, it is not clear what the role of splitting biochemical activities in this way might be. Using the biochemical reactions of CheA3, CheA4, and CheY6 as a model system, we developed a mathematical model and analyzed the response dynamics mediated by this split kinase. Repeating this analysis with a bifunctional HK and a conventional HK-RR pair featuring a separate phosphatase, we found that in contrast to these configurations, split kinases enable ultrasensitivity and bistability in the signal-response relationship. We show that these dynamical features are maintained under a wide parameter range, provided certain biochemical assumptions are met. These requirements indicate that the source of ultrasensitivity and bistability in split kinases is the inverse coupling between their kinase and phosphatase activities; i.e. the kinase activity cannot be increased without reducing the phosphatase activity and vice versa. Through measurements of phosphatase activity, we show that this condition is met in the *R. sphaeroides* system *in vitro*. These findings suggest that bacteria might be utilizing split kinases as a means of implementing ultrasensitivity and bistability in cellular decision making.

## Results

### Construction of a mathematical model of a split kinase

Since our aim is to study the general response dynamics that split kinases can mediate, we use the CheA3, CheA4, and CheY6 triplet as a model system and study its dynamics in isolation through *in vitro* experiments, numerical simulation and analytical approaches. We developed a mathematical model of the system and parameterized it with *in vitro* and *in vivo* measured kinetic rates and protein concentrations respectively (see *Methods* and Table 1). We then analyzed the response dynamics of the resulting model and its variants both through numerical simulations and deriving analytical solutions of steady state behavior using approximations and the chemical network theory [28], [29] (see*Methods* and *Text S1*). In the subsequent sections, we use the terms free CheA3 and free CheA3-P to indicate CheA3 species where the P1 domain is not interacting with the P4 domain of CheA4; *in vivo*, however, these species are expected to be always joined to the chemotaxis cluster by their P5 domains.

**Table 1:** Literature source and parameter values used in the analysis of the basic model.

Parameter	Description	Value	Unit	Ref
*k_1_*	On rate for binding of CheA3 and CheA4	100	(µM s−^1^)	[21] see also *Results*
*k_2_*	Off rate for binding of CheA3 and CheA4	10	s^−1^	[21] see also *Results*
*k_3_*	Forward rate for phosphorylation complex	1	(µM s)^−1^	[21]
*k_4_*	Reverse rate for phosphorylation complex	39	s^−1^	[21]
*k_5_*	*K_cat_* for phosphorylation of CheA3 by CheA4	varied	s^−1^	
*k_6_*	CheA3-P to CheY6 Phosphotransfer	0.775	(µM s)^−1^	[21]
*k_7_*	CheA3-P to CheY6 Reverse phosphotransfer	0.00283	(µM s)^−1^	[21]
*k_8_*	Autodephosphorylation	0.169	s^−1^	[7]
*k_9_*	Association of phosphatase assisted dephosphorylation complex	5.6	(µM s)^−1^	[48]
*k_10_*	Dissociation of phosphatase assisted dephosphorylation complex	0.04	s^−1^	[48]
*k_11_*	*K_cat_* for phosphatase assisted dephosphorylation	2.5	s^−1^	See *Methods*
[A3]_tot_	Total concentration of CheA3	90	µM	[7]
[A4]_tot_	Total concentration of CheA4	40	µM	[34]
[Y6]_tot_	Total concentration of CheY6	225	µM	[34]
[ATP]	Total concentration of ATP	1000	µM	

### The input-output relationship for the split kinase shows ultrasensitivity and bistability

**Figure 2:**
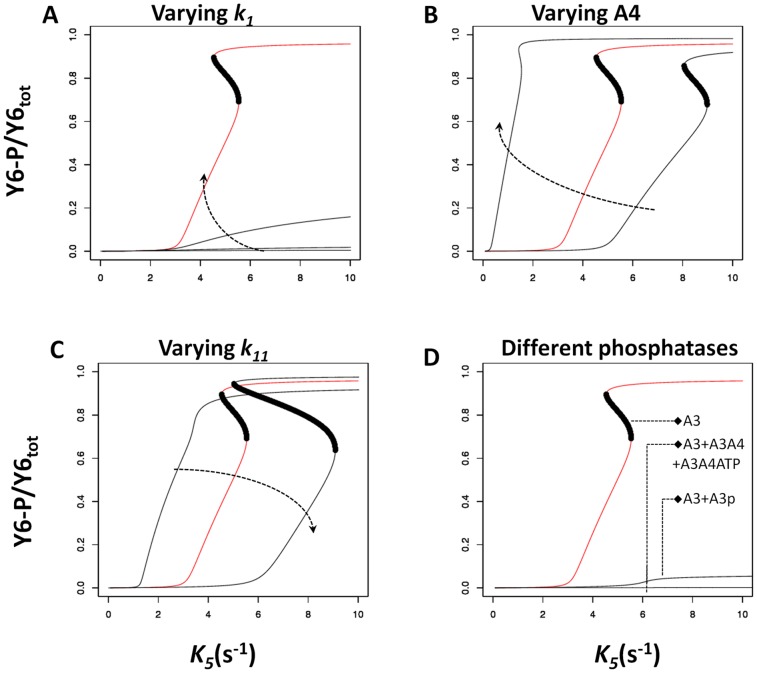
Effects of varying key parameters of the model and addition of different phosphatases. The x- and y-axis show the signal (*k_5_*) level and the corresponding steady state CheY6-P level respectively. Each panel shows a signal-response analysis for varying model parameters (A–C) or the inclusion of additional phosphatases (D). The results of the basic model are shown in red. Where present, the dark region indicates the region of unstable steady states and hence the presence of bistability. Arrows on panels A, B and C indicate increasing value of the changed parameter. (**A**) The on rate (*k_1_*) for CheA3:CheA4 complex formation was varied from basic model value [100(µMs)^−1^] to 10, 1, and 0.208. (**B**) Concentration of CheA4 was varied from 30 µM, 40 µM (basic model) and 80 µM. (**C**) The rate of CheA3 mediated dephosphorylation of CheY6-P (*k_11_*) was varied from 1 s^−1^, 2.5 s^−1^ (basic model) and 5s^−1^. (**D**) The basic model has free CheA3 as the sole phosphatase; the effect of having either CheA3-P or CheA3:CheA4 and CheA3:CheA4:ATP as additional phosphatases is shown. See also Figures S1, S2, S3, S4 for additional sensitivity analyses.

A primary property of interest for any signal transduction system is the signal-response relationship it implements [30]. To analyze the signal-response relationship in systems featuring a split kinase, we defined the system response as the steady state level of phosphorylated CheY6 (CheY6-P) at a given signal level, and derived this relationship for different parameters and biochemical assumptions (see *Methods*). This analysis revealed that when assuming free CheA3 as the sole phosphatase for CheY6-P, the system has a high potential for displaying ultrasensitivity and bistability (Figure 2 and Figures S1, S2, S3). Both of these dynamics result in a switch-like behavior; the response of the system is low until signal levels increase above a certain threshold, after which the response increases disproportionately to reach a high level (e.g. Figure 2A). In the case of bistability, the low and high response levels correspond to stable states of the system, separated by an unstable region, resulting in abrupt switching dynamics and hysteresis (i.e. the switching threshold is different depending on the past state of the system).

The *in vitro* and *in vivo* measured kinetic rates and protein concentrations from *R. sphaeroides* constitute “biologically meaningful” values that could be representative for two-component systems in general. To analyze the potential effects of these rates on the observed nonlinearity of the signal-response relationship, we have performed a sensitivity analysis by varying the base parameter values over a large range and quantifying the shape of the resulting signal-response curve (see *Methods*). This analysis shows that the level of ultrasensitivity in the signal-response relationship is most sensitive to the parameters controlling the complex formation between CheA3:CheA4 (*k_1_*) and the dephosphorylation of phosphorylated CheY6 (*k_9_* and *k_11_*) (Figure 2 and Figures S1, S2, S3). The association rate constant (*k_1_*/*k_2_*) we used in the basic model is approximately 500-fold higher than that measured *in vitro*, using purified *R. sphaeroides* proteins [21]. We still consider this high value “biologically relevant” as *in vivo* conditions can result in confining of split kinase components to small regions of the cell, resulting in much higher effective concentrations than are attainable under the *in vitro* conditions as used in [21]. For example, in *R. sphaeroides*, CheA3 and CheA4 localize to the cytoplasmic chemoreceptor cluster [23], which - using immunogold electron microscopy - is estimated to occupy less than 5% of the cross-sectional area of the cell [31]. Assuming a spherical shape for both the cell and this cluster, the volume of the latter could be estimated to be approximately 1% of the total cell volume. Thus, the effective concentrations of CheA3 and CheA4 in this cluster could be increased by as much as 100-fold, resulting in a significantly higher effective association rate constant than measured *in vitro* (up to 10,000 fold).

Besides parameter values, several modeling choices could also alter the finding of bistability and ultrasensitivity arising in a split kinase system. In particular, the basic model presented above assumes that free CheA3 is the sole phosphatase in the system (besides the intrinsic autodephosphorylation activity of CheY6-P). Relaxing this assumption and considering increasing phosphatase activity by the CheA3:CheA4 and CheA3:CheA4:ATP complexes (see *Text S1, section 1*), significantly reduced ultrasensitivity in the system (Figure 2D and S4). In contrast, the presence of ultrasensitivity was much more robust to increasing phosphatase activity by CheA3p (Figure 2D, S4 and S5). Another mechanistic choice in the modeling of the split kinase system is the fate of the CheA3:CheA4 complex after phosphorylation of CheA3. In the basic model analyzed in Figure 2, this is modeled as phosphorylation resulting in the dissociation of the complex and release of CheA4 and CheA3-P. An alternative would be that the CheA3:CheA4 complex remains intact post phosphorylation, resulting in a CheA3-P:CheA4 complex (see *Text S1, section 2*). When we assume the presence of CheA3-P:CheA4 complex that can phosphotransfer to CheY6, bistability was lost, but not ultrasensitivity (Figure S6). Finally, we found that including an additional (monofunctional, non-split) kinase in the model, as seen for example in *R. sphaeroides* CheA2 (see *Text S1, section 3*), does not affect the ultrasensitivity but can result in the loss of bistability (Figure S7).

It is important to note that the basic model and all of these variants arising from specific modeling choices are “nested” in the sense that the basic model can be recovered through appropriate choice of parameters (e.g. setting dephosphorylation activity of CheA3p very low). In line with this observation, we find that the basic model and all of the alternative structures discussed so far can be analytically shown to possess the “ability” to attain bistability (see *Methods*). More particularly, each of the chemical reaction systems arising from these models have the capacity for multiple steady states according to the higher deficiency theorem [32], [29]; i.e. these chemical systems permit bistability for some set of non-zero parameter values and under the assumption of mass action kinetics (see *Text S2*).

### Segregation of kinase and phosphatase activities allows ultrasensitivity and bistability

**Figure 3:**
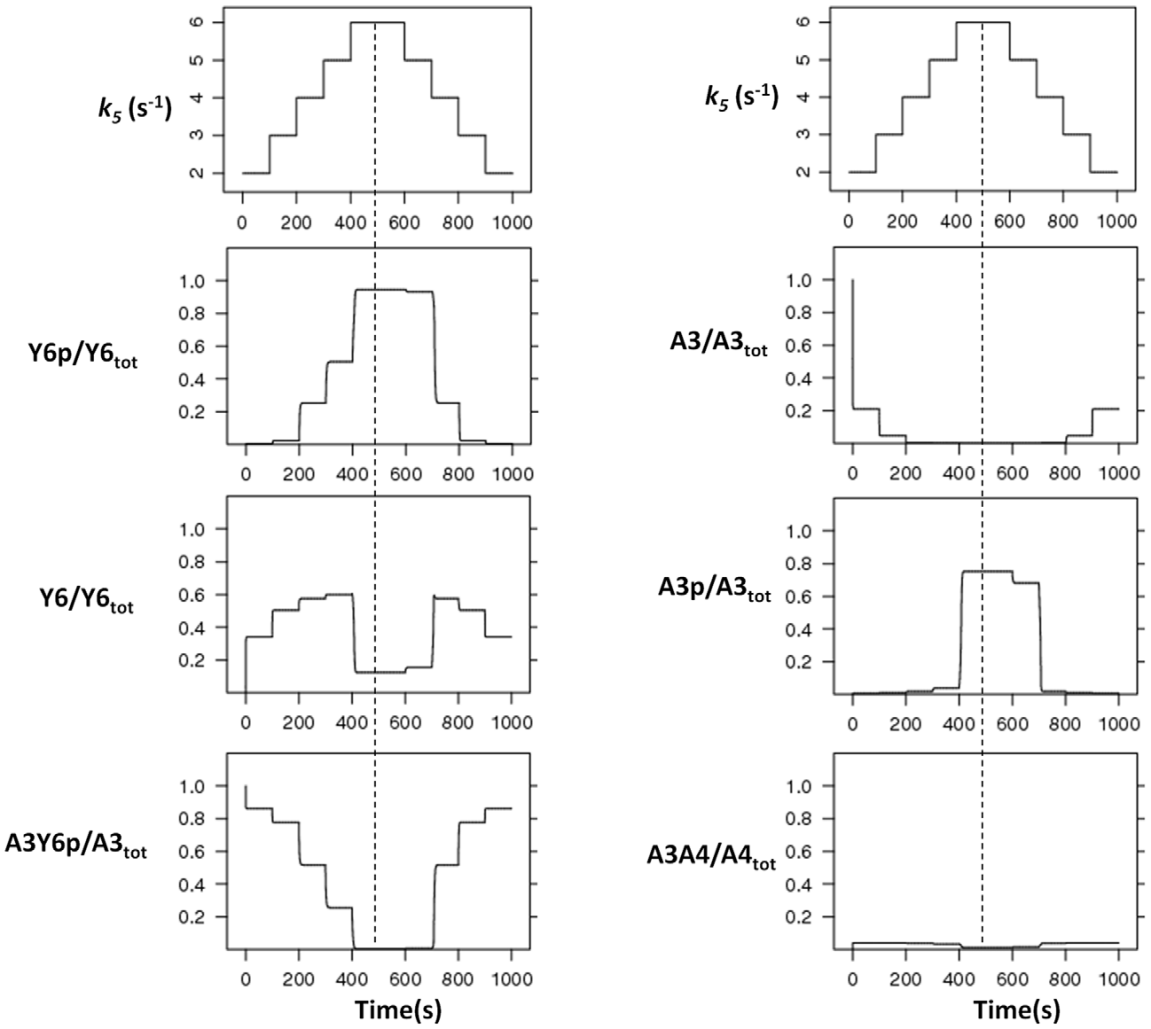
Time-course analyses. The model is simulated with increasing and decreasing signal levels (*k_5_*) in course of time. *k_5_* is increased from 2 to 6 and decreased in similar fashion at indicated time points (top most, left panel), and changes in each species were measured (as indicated on each panel). The dotted line represents the highest signal level, with equal signal steps on each side of it. The noted asymmetry around this line shows the presence of hysteresis in the system. The x- and y-axis represent time and species concentration respectively, where the latter is normalized by the appropriate total protein levels.

Taken together, these analyses suggest that the ability of a split kinase to mediate ultrasensitivity and bistability relates to the segregation of kinase and phosphatase activities. To better understand how this relates to ultrasensitivity and bistability, we simulated the time evolution of the system in the presence of step signals. As expected from the ultrasensitive signal-response relationship, system response (i.e. increase in free CheY6-P) was low for step-signals below the threshold and displayed a sudden large jump for step-signals crossing the threshold (Figure 3). Before the threshold, increasing signal levels resulted in an increase in the CheA3:CheY6-P complex, while the crossing of the threshold and subsequent increases in signal caused it to decrease. The reason for this behavior is that before the threshold there is enough free CheA3 in the system to bind and dephosphorylate the CheY6-P that is formed, while after crossing of the threshold there is no free CheA3 left in the system (Figure 3). These observations can be understood if we consider the cyclic nature of the reactions in this system as shown in Figure 1. The free CheA3 can be seen as a branching point in the system, with one branch leading to binding to CheA4 and ultimately to more CheY6 phosphorylation (phosphorylation branch), while the other leading to binding to CheY6-P and subsequent dephosphorylation (dephosphorylation branch). While the phosphorylation branch is regulated externally of the system by signals sensed by the cytoplasmic cluster (i.e. through altering *k3* and/or *k5*), the dephosphorylation branch is controlled internally by the covalent modification of CheY6. This results in a dynamical motif that is similar to that seen in metabolic branching points and that can embed ultrasensitivity [33]. The split kinase system can embed a high level of nonlinearity as it contains both an inverse coupling of the two branches themselves (via CheY6) and their regulation (via CheA3). At low signals, these two branches allow enough free CheA3 in the system so to result in equally fast phosphorylation and dephosphorylation of CheY6. As the signal increases, however, the rate of the phosphorylation branch increases, while at the same time shutting down the dephosphorylation branch. In other words, the phosphorylation and dephosphorylation branches are coupled inversely, such that the kinase activity cannot be increased without reducing the phosphatase activity and vice versa. These dynamics can be observed in Figure 3; the loss of free CheA3 in the system coincides with an abrupt increase in CheA3-P and CheY6-P, while the CheA3:CheA4 complex maintains a fast turnover. This dynamical picture also explains the parameter effects observed in Figure 2 (and Figures S1, S2, S3, S4). For example, the decrease in ultrasensitivity from the reduction of CheA3-CheA4 association rate constant (*k1*) can be explained by a slowing down of the phosphorylation branch. Similarly, the decrease in ultrasensitivity from the inclusion of additional phosphatase activity via species other than free CheA3 can be explained by its perturbing effects on the inverse coupling between the phosphorylation and dephosphorylation branches (Figure S4 and S5). It must also be noted that the total level of CheA4 in the cell allows additional (internal) control on the dynamics of the system (Figure 2B and Figure S3), through its effects on the phosphorylation branch.

To further test whether the inverse coupling of kinase and phosphatase activities through free CheA3 is the underpinning mechanism of ultrasensitivity, we considered dynamics in two alternative models where such coupling is missing; (i) a bifunctional HK that is not split, and (ii) a traditional HK that is neither bifunctional nor split, with a dedicated auxiliary phosphatase for the phosphorylated RR. An analytical treatment of the dynamics arising in the former scenario suggests that non-split bifunctional HKs (where the phosphorylated/non-phosphorylated HK acts as kinase/phosphatase on its cognate response regulator) gives rise to hyperbolic signal-response relationships and provides the system with robustness towards variations in component concentrations [9]. For the latter scenario (e.g. CheA-CheY-CheZ system found in the *E. coli* chemotaxis system) we developed a simplified model and solved it for the steady state levels of phosphorylated response regulator. We compared this analytical solution to that derived from a simplified model of a split kinase system (see *Text S1, section 4*). This analytical treatment shows that the latter displays a higher level of nonlinearity for the steady state expression of phosphorylated RR. More importantly, we find that of the three possible alternative structures - bifunctional and split, monofunctional and split, bifunctional and non-split - only the chemical reaction system arising from the bifunctional and split kinase have the capacity for multiple steady states according to the higher deficiency theorem [32], [29] (see *Text S3–6* for detailed results). Taken together, these analytical findings show that for bistable and ultrasensitive dynamics to be realized in a split kinase system, *both* bifunctionality of the HK and the splitting of these two functionalities (i.e. kinase and phosphatase activity) are needed.

### Experimental verification that free CheA3 is a better phosphatase than CheA3:CheA4

**Figure 4:**
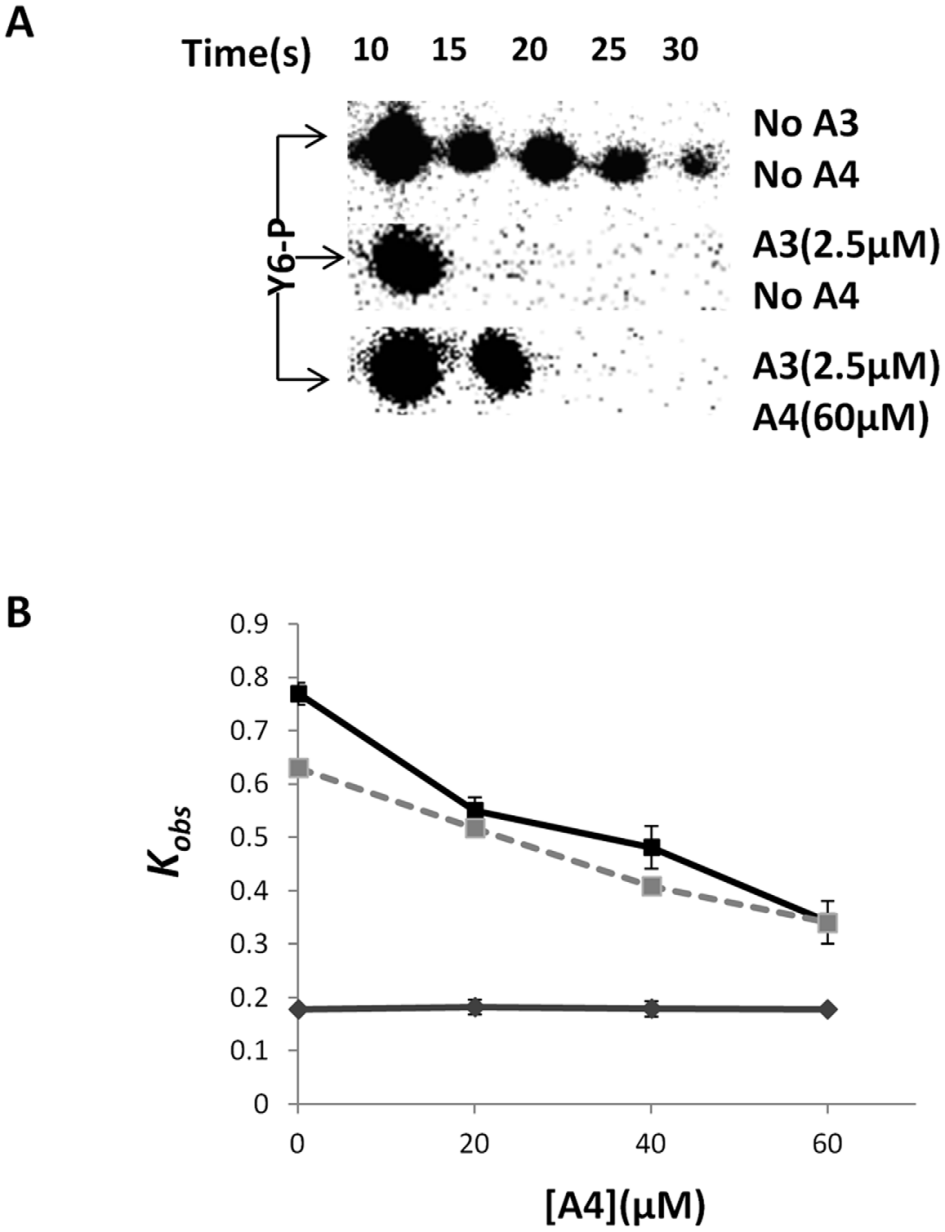
Measurement of CheY6-P dephosphorylation rates under different conditions (as indicated). An excess of CheY6 was phosphorylated using CheA3P1-P as phosphodonor. The phosphotransfer reaction was complete within 10 s of adding CheY6 to the reaction mixture. Subsequently the decay in CheY6-P levels was followed over time. (**A**) Phosphorimages showing the decay in CheY6-P levels over time. (**B**) Graph comparing the observed pseudo-first order rate constant (k*_obs_*) for CheY6-P dephosphorylation with and without CheA3 and CheA4. The values predicted by the modeling are shown with a dashed line, while the experimentally measured values are shown in black. Results from a control experiment (without CheA3 and solely CheA4) is shown in grey. Error bars show the standard error of the mean obtained from eight independent experiments.

As shown above, the ability of the split kinase to achieve both segregation and inverse coupling of kinase and phosphatase activities requires that free CheA3 is the predominant phosphatase with other CheA3 containing species (in particular CheA3:CheA4 and CheA3:CheA4:ATP) showing much lower phosphatase activity. Testing this requirement, or directly the level of ultrasensitivity *in vivo*, is complicated both by the presence of additional components in the system and our lack of knowledge of the signal identity in split kinase systems studied to date. As an alternative, and to achieve an approximate test of our theoretical understanding of split kinase response dynamics, we performed *in vitro* measurements of CheY6-P dephosphorylation in the presence of CheA3 and CheA4. In these experiments we used a purified phosphorylated P1 domain of CheA3 (CheA3P1-P) as the sole phosphodonor in the environment. As CheA3P1-P is known to lack phosphatase activity [7], this setup allows us to test directly the phosphatase activity of free CheA3 and the CheA3:CheA4 complex. If kinase and phosphatase activities are segregated into the complexed and free CheA3 respectively, these measurements should reveal a decrease of phosphatase activity with increasing CheA4 concentration, as this would sequester free CheA3 into the CheA3:CheA4 complex. In contrast, such an effect would be absent if the CheA3:CheA4 complex possessed the same level of phosphatase activity as free CheA3. We found evidence for such a decrease, with increasing CheA4 concentrations reducing the rate of CheA3 mediated dephosphorylation of CheY6-P (Figure 4 and Figure S8). To rule out the possibility of any interference from free CheA4, we have also confirmed the lack of dephosphorylation activity by CheA4 (Figure 4B). This observation qualitatively matches predictions from a specific model of this *in vitro* experimental setup where we assumed phosphatase activity to be restricted to only free CheA3 (see *Text S1* and Figure 4). These experimental findings strongly suggest that the CheA3:CheA4 complex has much lower phosphatase activity than free CheA3.

## Discussion

Two component signaling systems mediate many of the physiological responses of bacteria and display several conserved biochemical and structural features. Here, we analyzed how one such feature, the split kinase, affects response dynamics. Our theoretical treatment proved that the chemical reaction system arising from a bifunctional split kinase gives rise to the possibility of bistability, whereas systems arising from bifunctional, non-split and monofunctional, split kinases lack such capability (unless featuring dead-end complex formation [12]). Sampling the parameter space around kinetic rates and protein concentrations measured in (or estimated from) *R. sphaeroides*, we found that a split kinase system set in a “biologically relevant” parameter regime has potential for an ultrasensitive and bistable signal-response relationship. These nonlinear dynamics arise from the bifunctional and split nature of the kinase, which introduce a branching point into the system between phosphorylation and dephosphorylation reactions. Thus, the level of ultrasensitivity (and emergence of bistability) in the system is determined by the parameters and the biochemical mechanisms found in the reaction cycles linked to this branching point.

We found that the one crucial biochemical aspect enabling ultrasensitivity and bistability in the split kinase system is the predominant allocation of phosphatase activity to the free protein (rather than any of the complexes in the system). Using *in vitro* phosphotransfer assays in the CheA3-CheA4-CheY6 split kinase system isolated from *R. sphaeroides*, we found support for free CheA3 being the principal phosphatase in that system (Figure 4). It remains to be shown whether this system enables ultrasensitivity or bistability *in vivo*. The theoretical findings of this study suggest that the switch-like dynamics resulting from ultrasensitivity and bistability could be relevant in the physiological context of the CheA3-CheA4-CheY6 system, which is involved in the integration of cytoplasmic and extracellular signals for proper chemotaxis [7], [34]. It would be plausible for example, if the switching dynamics described here allowed cells to override external chemotaxis signals in favor of internal signals such as those related to metabolism, which could contribute to motility decisions [35]–[37]. As shown in Figure 2, several internal parameters of the system, including the total expression level of CheA4, allow control of the dynamics mediated through CheA3:CheA4 and might enable further tuning of such decision making mechanisms.

While our results highlight split kinases as a potential strategy for implementing ultrasensitivity in bacterial two-component systems, it is not the only one. Previous theoretical studies have found that ultrasensitivity can be achieved in phosphorelays [18], [19], in classical HK-RR systems embedding specific spatial dynamics [38] and in systems with bifunctional HKs, where unphosphorylated HKs and RR form a dead-end complex that is incapable of HK autophosphorylation [12], [39]. These findings suggest that there are several diverse structural, spatial and dynamics that are possible in bacterial two-component systems and that have the potential to enable nonlinear response dynamics. Our theoretical findings extend this list with split kinase systems. Further, we provide experimental support for a condition that increases their potential for generating ultrasensitivity and bistability. Such responses are known to be common in eukaryotes and can enable decision making at the cellular level [40]–[42]. Thus, it is perhaps not surprising that bacterial signaling systems harbor mechanisms to enable similar levels of ultrasensitivity.

Although rare, split kinases are found in several other bacteria. A recent study looking at CheAs identified 11 split CheAs (2.3%) versus 470 complete CheAs (97.7%) in fully sequenced non-redundant genomes [1]. In addition to these split CheAs, there is the potential for other HKs to be split where the HisKA (dimerization and histidine phosphotransfer) and the catalytic HATPase (histidine kinase ATPase) domains are found on separate proteins. *In vitro* studies of the osmosensing histidine kinase, EnvZ, have shown that it possible to split the HATPase and HisKA domains onto separate polypeptides whilst retaining their activity [43]. Interrogation of the SMART database reveals that out of the 42417 proteins containing HisKA domains (dimerization and histidine phosphotransferase), 1556 (3.66%) lack a HATPase (histidine kinase ATPase) domain (expect value<0.01), and of these, 711 (1.7%) have the phosphatase sequence motif (HE/DxxN/T) [44] and could therefore be split bi-functional kinases. The results presented here suggest that cells may use such split kinases to allow high sensitivity and bistability enabling switch-like physiological responses to environmental stimuli.

As the highly modular TCSNs are used by bacteria to control many of their physiological responses, it will be valuable to explore other mechanisms which can enable specific response dynamics in these systems and to determine the evolutionary drivers that were responsible for their emergence. This would increase our ability to better understand microbial signaling and exploit it in synthetic biology applications.

## Methods

### A mathematical model for a split kinase

To model the CheA3-CheA4-CheY6 split kinase system, we considered its dynamics in isolation of other cellular components. The reactions in this system that we have included in the “basic model” are (see also alternative reaction schemes shown in *Text S1*);
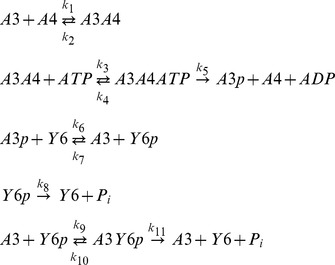
where *A3*, *A4*, *Y6* stand for CheA3, CheA4 and CheY6 respectively and the *-p* suffix represents phosphorylated forms of these proteins. Variant models which include additional CheY6-P de-phosphorylation reactions involving alternative phosphatases such as CheA3-P, and CheA3:CheA4 complex are shown in *supplementary text S1*, and their effects are analyzed in Figure 2D and S4. The above “basic model” reaction scheme can be used to derive a system of ordinary differential equations (ODEs), which describe the changes in concentrations of proteins over time;
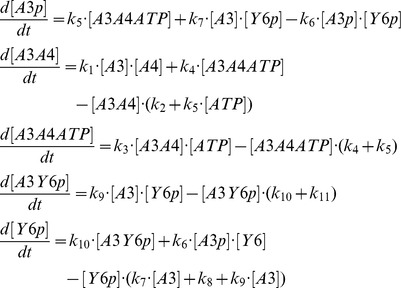
In addition, we have three conservation equations;
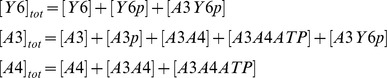
To analyze the behavior of the split kinase motif with increasing signal, we simulated the incoming signals from receptors as an increase in the autophosphorylation rate of the kinase (*k_5_*). The model was parameterized with data from literature (see Table 1). In the case of the dephosphorylation of CheY6-P by CheA3, we derived the relevant parameters (*k_9_*, *k_10_*, and *k_11_*) through fitting simulation data to previously published *in vitro* dephosphorylation measurements [7]. Fitting was done using a hybrid genetic algorithm (functions ga and fmincon from the MATLAB Global Optimization Toolbox).

We numerically integrated the model to derive time course and steady state signal-response relationships. The latter analysis gives the steady state CheY6-P level at a given signal (*k_5_*) where signal was taken as the rate of autophosphorylation of split kinase and allows deriving a so-called signal-response curve. This curve is found by numerically integrating the system to steady state at a fixed signal level and then numerically “following” this steady state (i.e. steady state CheY6-P level), while changing the signal. This analysis is equal to allowing the system to reach steady state under different signal values. Both time course and signal-response analyses were performed using the software packages XPPAUT (http://www.math.pitt.edu/~bard/xpp/xpp.html) and Oscill8 (http://oscill8.sourceforge.net/).

#### Sensitivity analysis

We have quantified the sensitivity of the shape of the signal-response curves to variations in each of the parameters from their described base values (Table 1) and in a biologically relevant range. For these analyses, we measured the “sigmoidality” of the signal-response curve, *RS*, as its maximum slope (*s_max_*) multiplied by the signal level at which this slope occurs (*k_5s_*) (i.e. *RS = k_5s_*•*s_max_*). This measure is similar to the “response coefficent”, which measures the slope between 90% and 10% saturation [33], but is better able to distinguish between hyperbolic and sigmoidal dose-response curves. For each parameter, we varied it in a wide range around its basic value and measured “sigmoidality” of the resulting dose-response curves, as well as the maximum response of the system (Figures S1, S2, S3). The same analysis is also applied for alternative models featuring additional phosphatase species (Figure S4).

#### Analytical comparison of different models

To perform a formal check for the potential of bistability in the different models (discussed in the main text and *Supplementary Information*), we have utilized the chemical network theory [28], [29]. This theory provides several analytical tests that can provide a definite answer on the possibility of existence of multiple stationary states in a given reaction network. We have applied these tests to the basic and alternative models we had devised using the Chemical Network Tool v2.2 (http://www.chbmeng.ohio-state.edu/~feinberg/crntwin/). The model files used with this tool and describing the chemical reaction systems, as well as the analytical results from the tool are provided as supplementary *Text S2–4*.

#### Plasmid and strains

See Table 2 for the plasmids and strains used. *E. coli* strains were grown in LB medium at 37°C. Antibiotics were used at concentrations of 100 µg ml−1 for ampicillin and 25 µg ml−1 for kanamycin, where needed. *E. coli* M15pRep4 cells were made competent using the calcium chloride technique [45]. Transformations were performed according to [46].

**Table 2:** Plasmids and strains used and the associated literature source.

Strains/plasmid	Description	Source/Reference
*E.coli* strain M15pREP4	Expression host containing pREP4; kanamycin resistant	Qiagen
pQE30	IPTG inducible expression vector. Introduces RGS(H)6 at the N terminus of the expressed protein. Confers ampicillin resistance	Qiagen
pQE60	IPTG inducible expression vector. Introduces RGS(H)6 at the C terminus of the expressed protein. Confers ampicillin resistance	Qiagen
pQE60A3P1	CheA3P1 expression plasmid. pQE60 derivative	[7]
pQEY6	CheY6 expression plasmid. pQE30 derivative	[24]
pQEA3	CheA3 expression plasmid. pQE30 derivative	[21]
pQEA4	CheA4 expression plasmid. pQE30 derivative	[21]

#### Protein purification

His tagged *R. sphaeroides* CheA3, CheA4, CheA3P1 and CheY6 proteins were purified as described previously [47]. Protein purity and concentration was measured as described in [24]. Purified proteins were stored at −20°c.

#### Preparation of CheA3P1-^32^P

CheA3P1 was phosphorylated using [γ-^32^P] ATP and CheA4 and purified as described before with the following modifications [7]. Proteins were phosphorylated in reactions performed at 20°C in phosphotransfer buffer (50 mM Tris HCl, 10% (v/v) glycerol, 5 mM MgCl_2_, 150 mM NaCl, 50 mM KCl, 1 mM DTT, pH 8.0). The final reaction volumes were 2 ml. For production of CheA3P1-^32^P, reaction mixtures contained 300 µM CheA3P1 and 20 µM CheA4. Reactions were initiated by addition of 2 mM [γ-^32^P] ATP (specific activity 14.8 GBq mmol^−1^; PerkinElmer). After 1 hour incubation, samples were purified by using Ni-NTA columns (Qiagen) as described previously for unphosphorylated His-tagged CheA3 [47]. This purification step removed the unincorporated ATP and also removed the CheA4 protein from the CheA3P1-^32^P preparation. Purified proteins were stored at −20°C.

### Measurement of CheY6-P dephosphorylation rate

Assays were performed at 20°C in phosphotransfer buffer. Purified CheA3P1-^32^P was used as the phosphodonor. An excess of CheY6 (100 µM) was added to 30 µM of purified CheA3P1-^32^P in the presence of 2.5 µM CheA3 and 0–60 µM CheA4. Following the addition of CheY6, reaction aliquots of 10 µl were taken at the indicated time points and quenched immediately in 10 µl of 2 X SDS-PAGE loading dye(7.5% (w/v) SDS, 90 mM EDTA, 37.5 mM Tris HCl, 37.5% glycerol, 3% (v/v) β- mercaptoethanol, pH 6.8). Quenched samples were analyzed using SDS-PAGE and phosphorimaging as described previously [24].

## Supporting Information

Figure S1 The sensitivity of the signal response curve “sigmoidality” to parameter changes. The “sigmoidality” of the signal-response curve, *RS*, is measured as its maximum slope (*s_max_*) multiplied by the signal level at which this slope occurs (*k_5s_*) (i.e. *RS = k_5s_*•*s_max_*). On each panel, the y-axis shows the ratio of *RS*, resulting from models with different values of a specific parameter, to that resulting from the basic model. x-axis shows the ratio of this parameter value to its corresponding value in the basic model. Data points in red indicates presence of bistability in the signal-response relationship. Note the log scale on both axes.
(TIF)

Figure S2 The sensitivity of the maximum phosphorylation level of CheY6 to parameter changes. On each panel, the y-axis shows the ratio of the maximal CheY6 phosphorylation, resulting from models with different values of a specific parameter, to that resulting from the basic model. x-axis shows the ratio of this parameter value to its corresponding value in the basic model. Data points in red indicates presence of bistability in the signal-response relationship. Note the log scale on both axes.
(TIF)

Figure S3 The sensitivity of the signal response curve “sigmoidality” to changes in the concentration of CheA3 (**A**) and CheA4 (**B**). The “sigmoidality” of the signal-response curve, *RS*, is measured as its maximum slope (*s_max_*) multiplied by the signal level at which this slope occurs (*k_5s_*) (i.e. *RS = k_5s_* • *s_max_*). On panel A (B), the y-axis shows the ratio of *RS*, resulting from models with different values of CheA3 (CheA4) concentration, to that resulting from the basic model. x-axis shows the ratio of this concentration to its corresponding value in the basic model. Data points in red indicates presence of bistability in the signal-response relationship. The sensitivity of the maximum phosphorylation level of CheY6 to changes in the concentration of CheA3 (**C**) and CheA4 (**D**). On panel C (D), the y-axis shows the ratio of the maximal CheY6 phosphorylation, resulting from models with different values of CheA3 (CheA4) concentration, to that resulting from the basic model. x-axis shows the ratio of this concentration to its corresponding value in the basic model. Data points in red indicates presence of bistability in the signal-response relationship. Note the log scale on both axes on all panels.
(TIF)

Figure S4 Analysis of signal-response relationship, in an alternative model considering phosphatase activity from additional species (see Supplementary Information, section 1). (**A**) Signal-response curves resulting from a model where both CheA3:CheA4 and CheA3:CheA4:ATP are considered to have phosphatase activity in addition to CheA3. For comparison, signal-response curve from the basic model is shown in red. Where present, the dark region indicates the region of unstable steady states and hence the presence of bistability. The different curves correspond to increasing levels of phosphatase activity (shown with the arrow) from the additional species. Phosphatase activity is varied in the same way for both CheA3:CheA4 and CheA3:CheA4:ATP by assuming that *k_on_* and *k_cat_* for these species are the same (i.e. *k_12_* = *k_15_* and *k_14_* = *k_17_*) and by varying one set of rates simultaneously. The ratio between these rates (*k_12_* and *k_14_*) to their corresponding values for CheA3 (*k_9_* and *k_11_*) is shown on the x-axis of panel C. (**B**) Signal-response curves resulting from a model where CheA-P is considered to have phosphatase activity in addition to CheA3. For comparison, signal-response curve from the basic model is shown in red. Where present, the dark region indicates the region of unstable steady states and hence the presence of bistability. The different curves correspond to increasing levels of phosphatase activity (shown with the arrow) from CheA3-P. Phosphatase activity is varied by changing both *k_on_* and *k_cat_* for CheA3-P (i.e. *k_18_* and *k_20_*) simultaneously. The ratio between these rates (*k_18_* and *k_20_*) to their corresponding values for CheA3 (*k_9_* and *k_11_*) is shown on the x-axis of panel D. (**C**) The sensitivity of the signal response curve “sigmoidality” to increasing phosphatase activity from CheA3:CheA4 and CheA3:CheA4:ATP. The “sigmoidality” of the signal-response curve, *RS*, is measured as its maximum slope (*s_max_*) multiplied by the signal level at which this slope occurs (*k_5s_*) (i.e. *RS = k_5s_* • *s_max_*). y-axis shows the ratio of *RS*, resulting from models with increasing phosphatase activity by additional species, to that of resulting from the basic model. X-axis shows the ratio of kinetic rates governing phosphatase activity (*k_12_* and *k_14_*) to those in the basic model (*k_9_* and *k_11_*). Data points in red indicates presence of bistability in the signal-response relationship. (**D**) The sensitivity of the signal response curve “sigmoidality” to increasing phosphatase activity from CheA3-P. The “sigmoidality” of the signal-response curve, *RS*, is measured as its maximum slope (*s_max_*) multiplied by the signal level at which this slope occurs (*k_5s_*) (i.e. *RS = k_5s_* • *s_max_*). Y-axis shows the ratio of RS, resulting from models with increasing phosphatase activity by additional species, to that of resulting from the basic model. x-axis shows the ratio of kinetic rates governing phosphatase activity (*k_18_* and *k_20_*) to those in the basic model (*k_9_* and *k_11_*). Data points in red indicates presence of bistability in the signal-response relationship. Note the log scale on both axes in panels C and D.
(TIF)

Figure S5 Time-course analysis using an alternative model where both CheA3:CheA4 and CheA3:CheA4:ATP are considered to have phosphatase activity in addition to CheA3 (see Supplementary Information, section 1). The model is simulated with increasing and decreasing signal levels (*k_5_*) in course of time. *k_5_* is increased from 2 to 6 and decreased in similar fashion at indicated time points (top most, left panel), and changes in each species were measured (as indicated on each panel). The x- and y-axis represent time and species concentration respectively, where the latter is normalized by the appropriate total protein levels.
(TIF)

Figure S6 Signal-response curves resulting from an alternative model that allows for the possibility that phosphorylated CheA3 remains in complex with CheA4 and that this CheA3p:CheA4 complex is also capable of acting as phosphatase towards CheY6p (see Supplementary Information, section 2). The y-axis shows steady state Y6-P level normalised by total Y6, while x-axis shows signal (*k_5_*) level. Where present, a dark region indicates the region of unstable steady states and hence the presence of bistability. (a) The signal-response curve from the basic model (included for comparison). (b) Signal-response curve from the alternative model and simulating signal level through changing both *k′_5_* and *k_5_* simultaneously. (c) Signal-response curve from the alternative model and simulating signal level through changing *k_5_*, while *k′_5_* = 0.1 s−1.
(TIF)

Figure S7 Analysis of signal-response relationship, in an alternative model considering additional kinase activity (see Supplementary Information, section 3). (**A**) Signal-response curves resulting from a model where additional kinase activity (from CheA2) is considered. For comparison, the signal-response curve from the basic model is shown in red. Where present, the dark region indicates the region of unstable steady states and hence the presence of bistability. The different curves correspond to increasing levels of autophosphorylation rates for CheA2 (i.e. increasing background signalling through CheA2). (**B**) The sensitivity of the signal-response “sigmoidality” with increasing background kinase activity (from CheA2). The “sigmoidality” of the signal-response curve, *RS*, is measured as its maximum slope (*s_max_*) multiplied by the signal level at which this slope occurs (*k_5s_*) (i.e. *RS = k_5s_* • *s_max_*). y-axis shows the ratio of *RS*, resulting from models with increasing background kinase activity (k*_5_) to that of the case where such activity is minimal (i.e. k*_5_∼0). Data points in red indicates presence of bistability in the signal-response relationship. Note the log scale on both axes.
(TIF)

Figure S8 CheY6-P dephosphorylation time course data (circles) along with the fitted first-order exponential decay curves (red line) and simulated data (black line). The exponential fits are used to derive an estimate for overall CheY6p dephosphorylation rate (*k_obs_*), which are shown in Figure 4.
(TIF)

Table S1 Parameter values used for the models with additional phosphatases.
(PDF)

Table S2 Parameter values used for the models with alternative reaction scheme.
(PDF)

Table S3 Parameter values used for the models with additional kinase.
(PDF)

Table S4 Parameter values used for the model of the *in vitro* experimental system.
(PDF)

Text S1 Supplementary information on alternative models and their analyses.
(PDF)

Text S2 Results of the analytical analysis of the basic model. The file contains the reaction system considered and the report produced with the Chemical Network Tool v2.2 (http://www.chbmeng.ohio-state.edu/~feinberg/crntwin/).
(DOC)

Text S3 Results of the analytical analysis of a model with a monofunctional kinase and a separate phosphatase. The file contains the reaction system considered and the report produced with the Chemical Network Tool v2.2 (http://www.chbmeng.ohio-state.edu/~feinberg/crntwin/).
(DOC)

Text S4 Results of the analytical analysis of a model with a monofunctional kinase. The file contains the reaction system considered and the report produced with the Chemical Network Tool v2.2 (http://www.chbmeng.ohio-state.edu/~feinberg/crntwin/).
(DOC)

Text S5 Results of the analytical analysis of a model with a bifunctional, non-split kinase. The file contains the reaction system considered and the report produced with the Chemical Network Tool v2.2 (http://www.chbmeng.ohio-state.edu/~feinberg/crntwin/).
(DOC)

Text S6 Results of the analytical analysis of a model with a monofunctional, split kinase. The file contains the reaction system considered and the report produced with the Chemical Network Tool v2.2 (http://www.chbmeng.ohio-state.edu/~feinberg/crntwin/).
(DOC)
