# Supplementary figures and images for: Split Histidine Kinases Enable Ultrasensitivity and Bistability in Two-Component Signaling Networks
Source: PLoS Comput Biol. 2013 Mar 7;9(3):e1002949. doi: 10.1371/journal.pcbi.1002949 (PMC3591291; doi:10.1371/journal.pcbi.1002949)

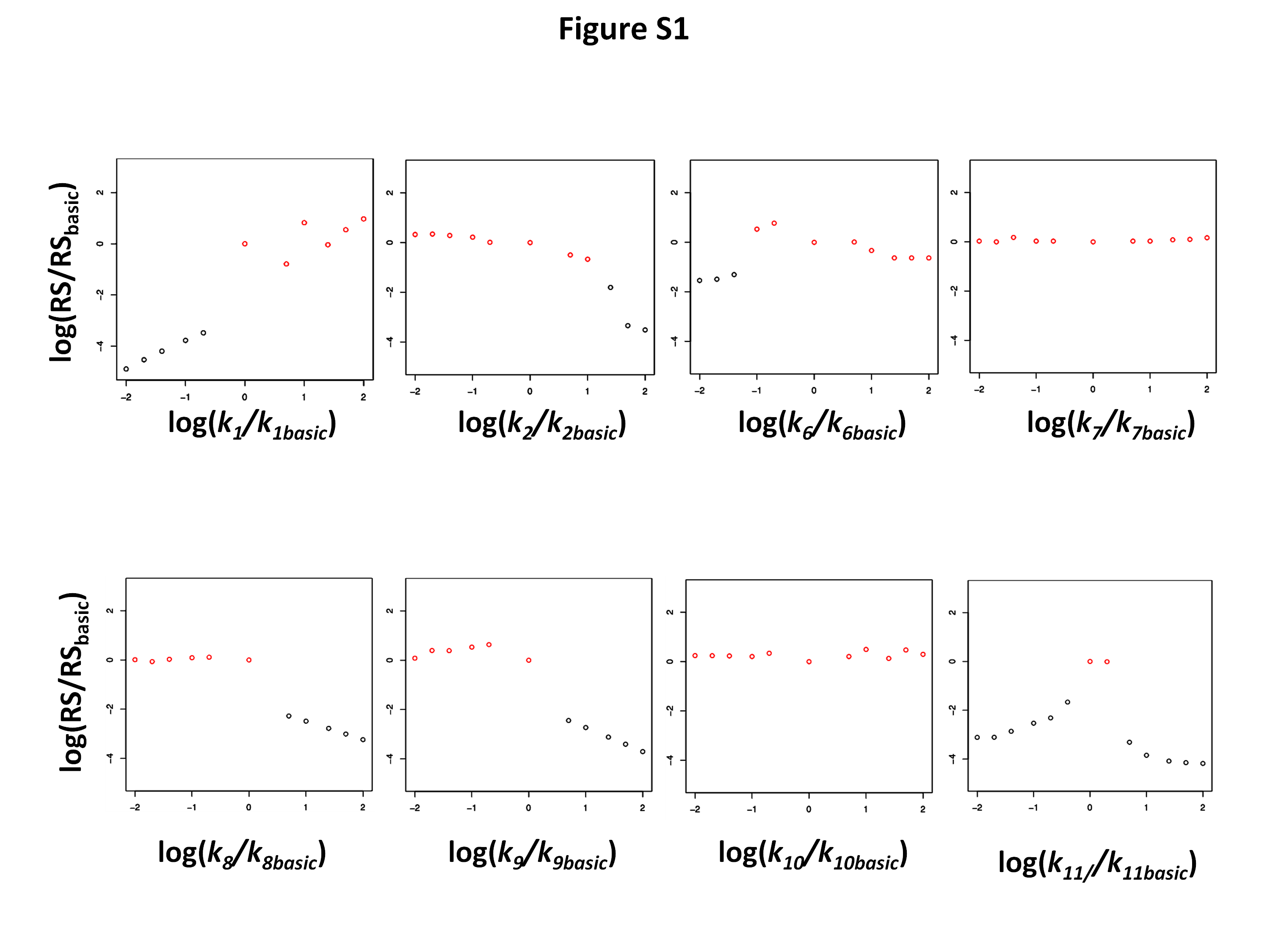

Supplement: Figure S1 — The sensitivity of the signal response curve “sigmoidality” to parameter changes. The “sigmoidality” of the signal-response curve, RS, is measured as its maximum slope (smax) multiplied by the signal level at which this slope occurs (k5s) (i.e. RS = k5s•smax). On each panel, the y-axis shows the ratio of RS, resulting from models with different values of a specific parameter, to that resulting from the basic model. x-axis shows the ratio of this parameter value to its corresponding value in the basic model. Data points in red indicates presence of bistability in the signal-response relationship. Note the log scale on both axes. (TIF) [file pcbi.1002949.s001.tif]

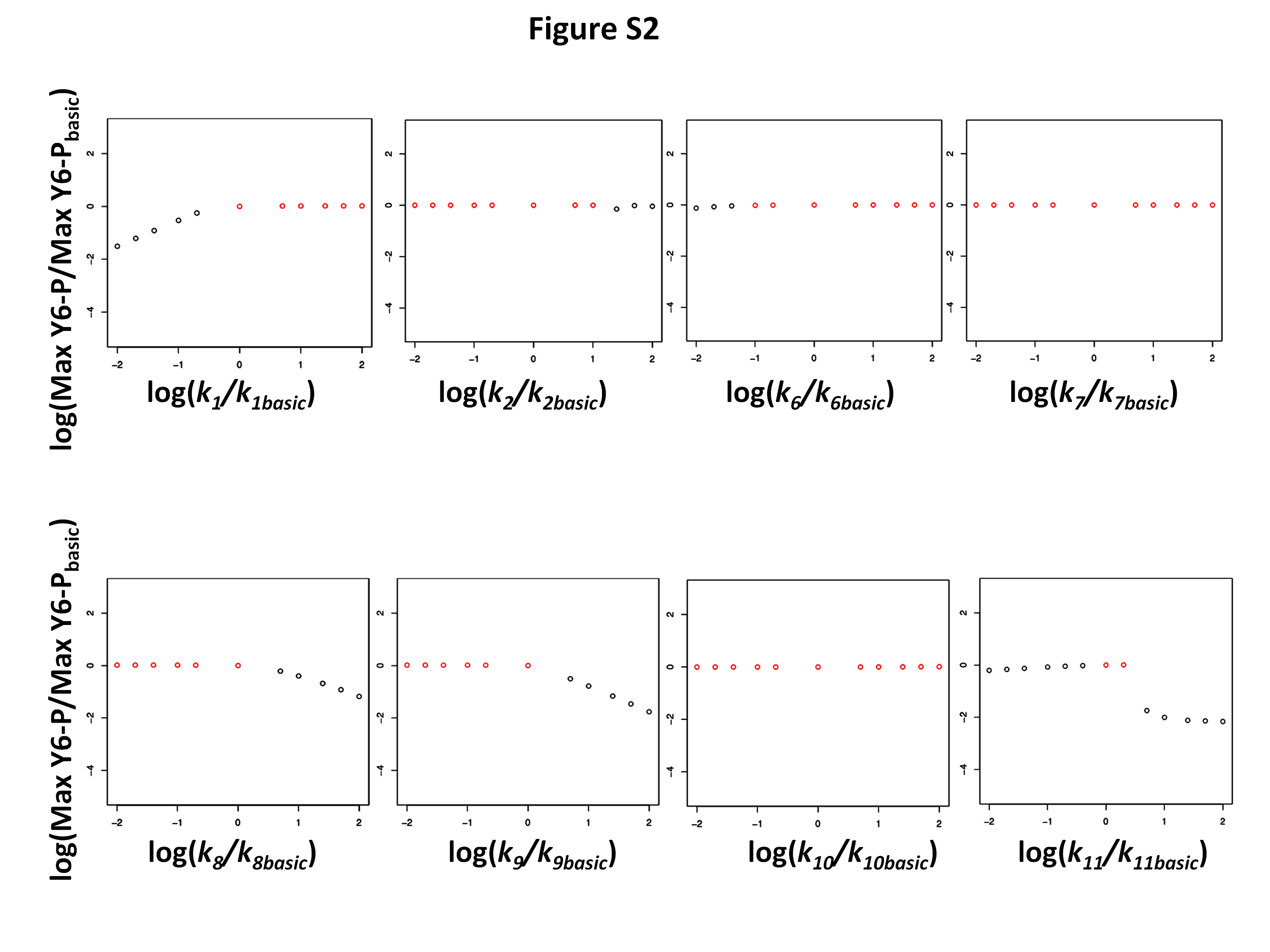

Supplement: Figure S2 — The sensitivity of the maximum phosphorylation level of CheY6 to parameter changes. On each panel, the y-axis shows the ratio of the maximal CheY6 phosphorylation, resulting from models with different values of a specific parameter, to that resulting from the basic model. x-axis shows the ratio of this parameter value to its corresponding value in the basic model. Data points in red indicates presence of bistability in the signal-response relationship. Note the log scale on both axes. (TIF) [file pcbi.1002949.s002.tif]

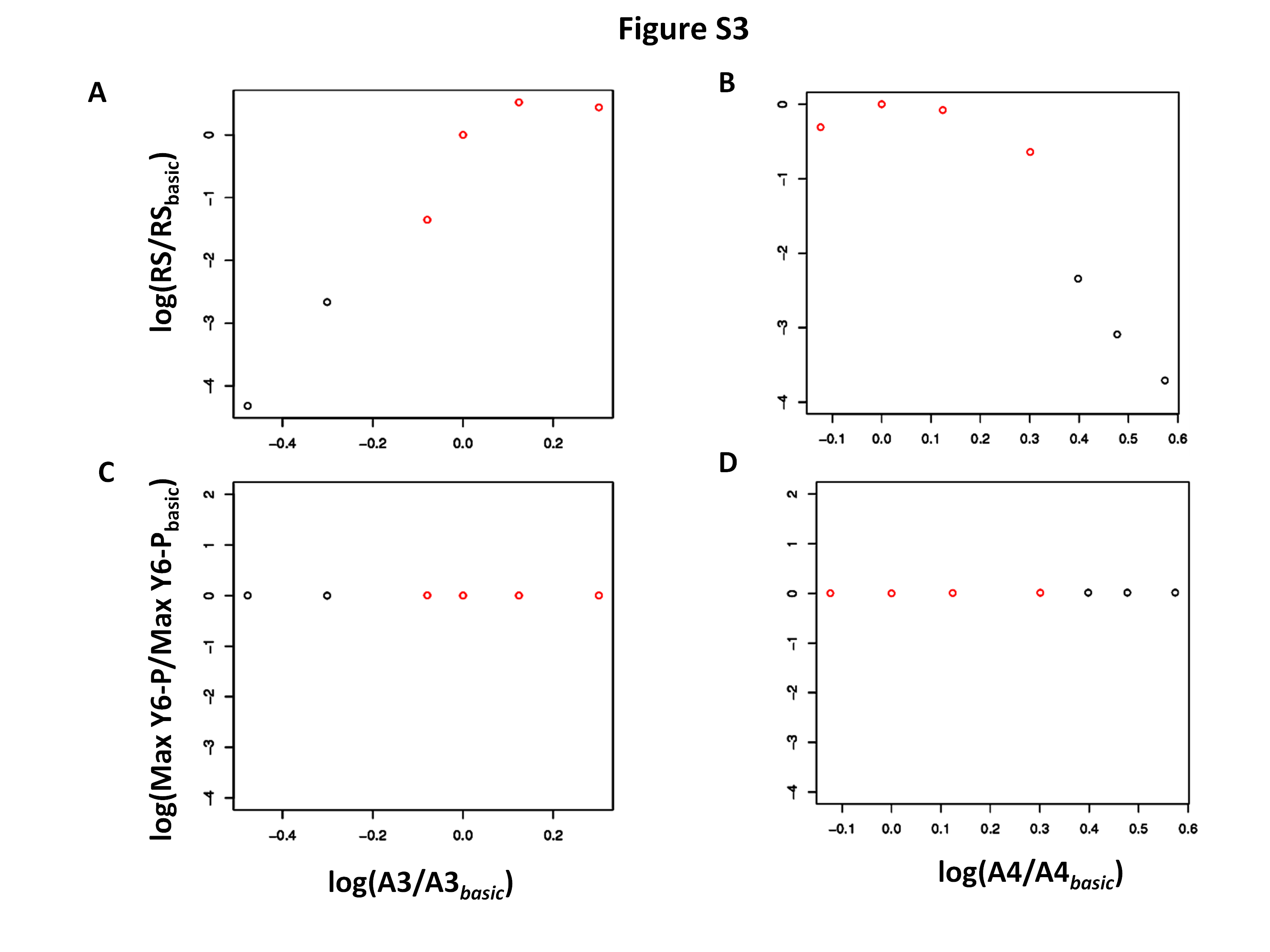

Supplement: Figure S3 — The sensitivity of the signal response curve “sigmoidality” to changes in the concentration of CheA3 (A) and CheA4 (B). The “sigmoidality” of the signal-response curve, RS, is measured as its maximum slope (smax) multiplied by the signal level at which this slope occurs (k5s) (i.e. RS = k5s • smax). On panel A (B), the y-axis shows the ratio of RS, resulting from models with different values of CheA3 (CheA4) concentration, to that resulting from the basic model. x-axis shows the ratio of this concentration to its corresponding value in the basic model. Data points in red indicates presence of bistability in the signal-response relationship. The sensitivity of the maximum phosphorylation level of CheY6 to changes in the concentration of CheA3 (C) and CheA4 (D). On panel C (D), the y-axis shows the ratio of the maximal CheY6 phosphorylation, resulting from models with different values of CheA3 (CheA4) concentration, to that resulting from the basic model. x-axis shows the ratio of this concentration to its corresponding value in the basic model. Data points in red indicates presence of bistability in the signal-response relationship. Note the log scale on both axes on all panels. (TIF) [file pcbi.1002949.s003.tif]

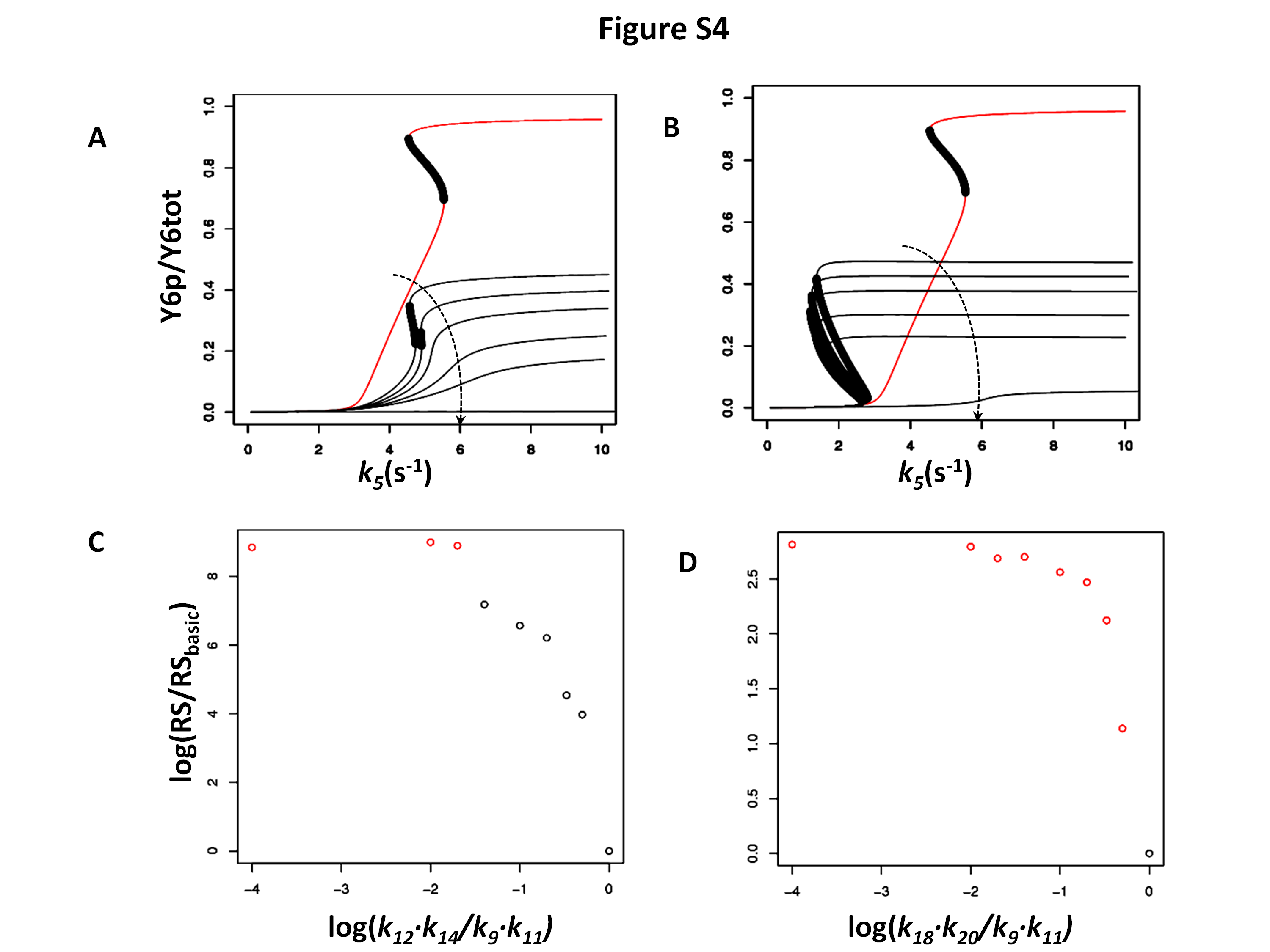

Supplement: Figure S4 — Analysis of signal-response relationship, in an alternative model considering phosphatase activity from additional species (see Supplementary Information, section 1). (A) Signal-response curves resulting from a model where both CheA3:CheA4 and CheA3:CheA4:ATP are considered to have phosphatase activity in addition to CheA3. For comparison, signal-response curve from the basic model is shown in red. Where present, the dark region indicates the region of unstable steady states and hence the presence of bistability. The different curves correspond to increasing levels of phosphatase activity (shown with the arrow) from the additional species. Phosphatase activity is varied in the same way for both CheA3:CheA4 and CheA3:CheA4:ATP by assuming that kon and kcat for these species are the same (i.e. k12 = k15 and k14 = k17) and by varying one set of rates simultaneously. The ratio between these rates (k12 and k14) to their corresponding values for CheA3 (k9 and k11) is shown on the x-axis of panel C. (B) Signal-response curves resulting from a model where CheA-P is considered to have phosphatase activity in addition to CheA3. For comparison, signal-response curve from the basic model is shown in red. Where present, the dark region indicates the region of unstable steady states and hence the presence of bistability. The different curves correspond to increasing levels of phosphatase activity (shown with the arrow) from CheA3-P. Phosphatase activity is varied by changing both kon and kcat for CheA3-P (i.e. k18 and k20) simultaneously. The ratio between these rates (k18 and k20) to their corresponding values for CheA3 (k9 and k11) is shown on the x-axis of panel D. (C) The sensitivity of the signal response curve “sigmoidality” to increasing phosphatase activity from CheA3:CheA4 and CheA3:CheA4:ATP. The “sigmoidality” of the signal-response curve, RS, is measured as its maximum slope (smax) multiplied by the signal level at which this slope occurs (k5s) (i.e. RS = k5s • smax). [file pcbi.1002949.s004.tif]

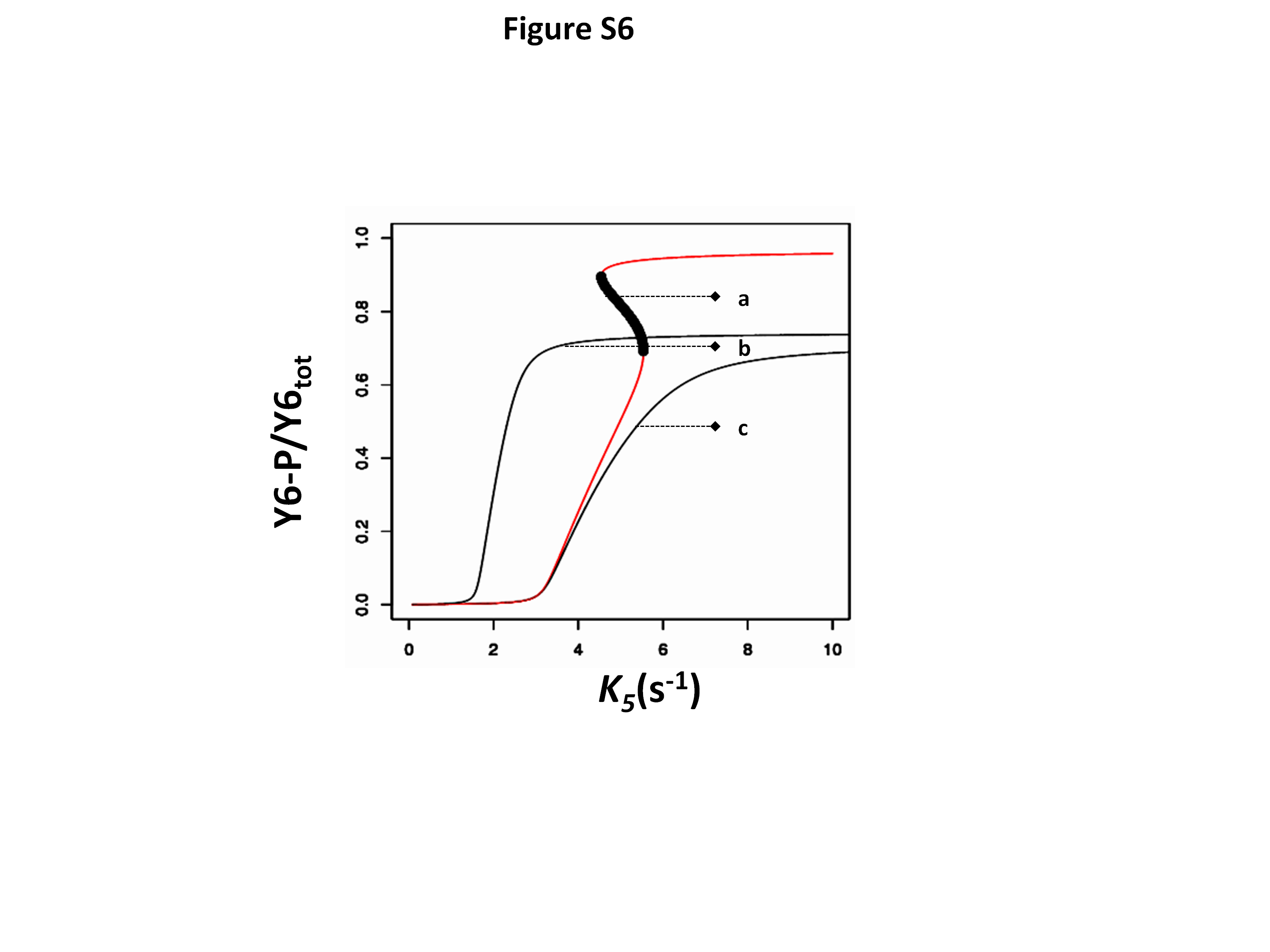

Supplement: Figure S6 — Signal-response curves resulting from an alternative model that allows for the possibility that phosphorylated CheA3 remains in complex with CheA4 and that this CheA3p:CheA4 complex is also capable of acting as phosphatase towards CheY6p (see Supplementary Information, section 2). The y-axis shows steady state Y6-P level normalised by total Y6, while x-axis shows signal (k5) level. Where present, a dark region indicates the region of unstable steady states and hence the presence of bistability. (a) The signal-response curve from the basic model (included for comparison). (b) Signal-response curve from the alternative model and simulating signal level through changing both k′5 and k5 simultaneously. (c) Signal-response curve from the alternative model and simulating signal level through changing k5, while k′5 = 0.1 s−1. (TIF) [file pcbi.1002949.s006.tif]

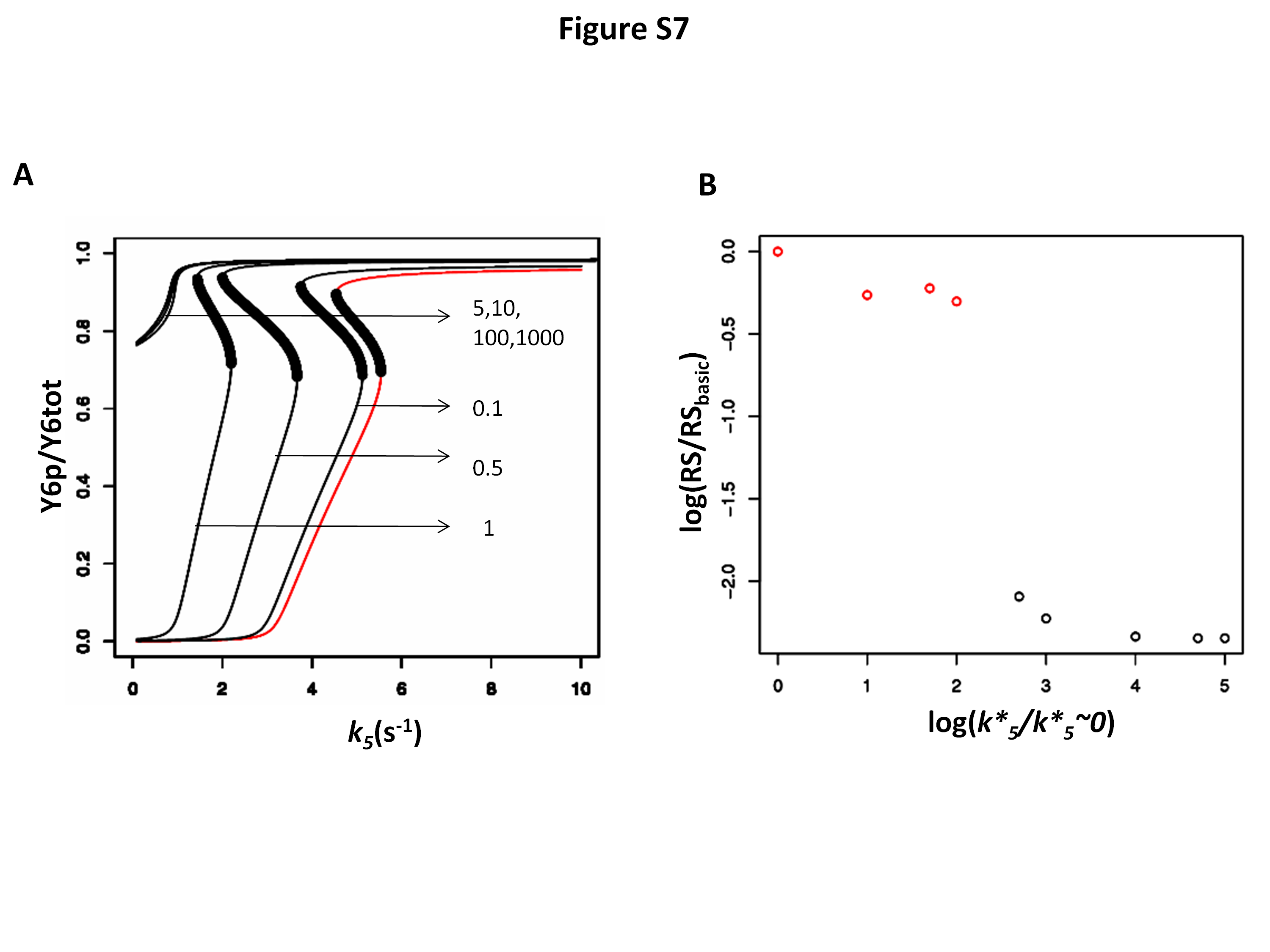

Supplement: Figure S7 — Analysis of signal-response relationship, in an alternative model considering additional kinase activity (see Supplementary Information, section 3). (A) Signal-response curves resulting from a model where additional kinase activity (from CheA2) is considered. For comparison, the signal-response curve from the basic model is shown in red. Where present, the dark region indicates the region of unstable steady states and hence the presence of bistability. The different curves correspond to increasing levels of autophosphorylation rates for CheA2 (i.e. increasing background signalling through CheA2). (B) The sensitivity of the signal-response “sigmoidality” with increasing background kinase activity (from CheA2). The “sigmoidality” of the signal-response curve, RS, is measured as its maximum slope (smax) multiplied by the signal level at which this slope occurs (k5s) (i.e. RS = k5s • smax). y-axis shows the ratio of RS, resulting from models with increasing background kinase activity (k*5) to that of the case where such activity is minimal (i.e. k*5∼0). Data points in red indicates presence of bistability in the signal-response relationship. Note the log scale on both axes. (TIF) [file pcbi.1002949.s007.tif]

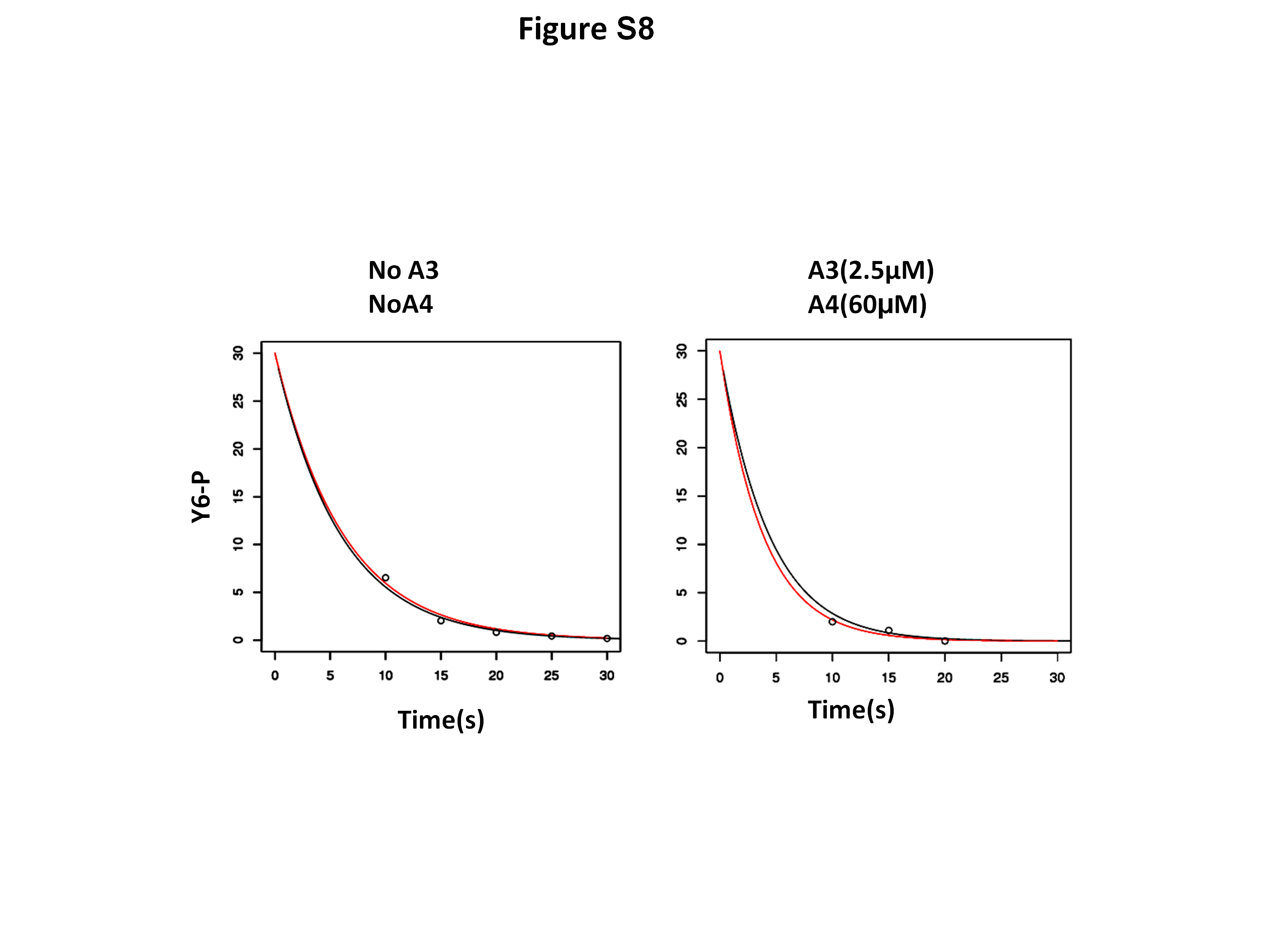

Supplement: Figure S8 — CheY6-P dephosphorylation time course data (circles) along with the fitted first-order exponential decay curves (red line) and simulated data (black line). The exponential fits are used to derive an estimate for overall CheY6p dephosphorylation rate (kobs), which are shown in Figure 4. (TIF) [file pcbi.1002949.s008.tif]
