# Supplementary material for: Split Histidine Kinases Enable Ultrasensitivity and Bistability in Two-Component Signaling Networks
Source: PLoS Comput Biol. 2013 Mar 7;9(3):e1002949. doi: 10.1371/journal.pcbi.1002949 (PMC3591291; doi:10.1371/journal.pcbi.1002949)
Supplement: Table S1 — Parameter values used for the models with additional phosphatases. (PDF) [file pcbi.1002949.s009.pdf]

Table S1

Parameter	Description	Value	Unit
k_9	Association of phosphatase (CheA3) assisted dephosphorylation complex	5.6	$(\mu Ms)^{-1}$
k_{12}	Association of phosphatase (CheA3CheA4) assisted dephosphorylation complex	5.6	$(\mu Ms)^{-1}$
k_{15}	Association of phosphatase (CheA3CheA4ATP) assisted dephosphorylation complex	5.6	$(\mu Ms)^{-1}$
k_{18}	Association of phosphatase (CheA3-P) assisted dephosphorylation complex	5.6	$(\mu Ms)^{-1}$
k_{10}	Dissociation of phosphatase (CheA3) assisted dephosphorylation complex	0.04	s^{-1}
k_{13}	Dissociation of phosphatase assisted (CheA3CheA4) dephosphorylation complex	0.04	s^{-1}
k_{16}	Dissociation of phosphatase (CheA3CheA4ATP) assisted dephosphorylation complex	0.04	s^{-1}
k_{19}	Dissociation of phosphatase (CheA3-P) assisted dephosphorylation complex	0.04	s^{-1}
k_{11}	K_{cat} for phosphatase (CheA3) assisted dephosphorylation	2.5	s^{-1}
k_{14}	K_{cat} for phosphatase (CheA3CheA4) assisted dephosphorylation	2.5	s^{-1}
k_{17}	K_{cat} for phosphatase (CheA3CheA4ATP) assisted dephosphorylation	2.5	s^{-1}
k_{20}	K_{cat} for phosphatase (CheA3-P) assisted dephosphorylation	2.5	s^{-1}
