# Supplementary material for: Split Histidine Kinases Enable Ultrasensitivity and Bistability in Two-Component Signaling Networks
Source: PLoS Comput Biol. 2013 Mar 7;9(3):e1002949. doi: 10.1371/journal.pcbi.1002949 (PMC3591291; doi:10.1371/journal.pcbi.1002949)
Supplement: Table S2 — Parameter values used for the models with alternative reaction scheme. (PDF) [file pcbi.1002949.s010.pdf]

Table S2

Parameter	Description	Value	Unit
k_1	On rate for binding of CheA3 and CheA4	100	(μMs-^{1})
k'_1	On rate for binding of CheA3-P and CheA4	100	(μMs-^{1})
k_2	Off rate for binding of CheA3 and CheA4	10	s^{-1}
k'_2	Off rate for binding of CheA3-P and CheA4	10	s^{-1}
k_5	K_{cat} for phosphorylation of CheA3 by CheA4	varied	s^{-1}
k'_5	K_{cat} for phosphorylation of CheA3 by CheA4	varied	s^{-1}
k'_6	CheA4/CheA3-P to CheY6 Phosphotransfer	0.775	(µMs) -1
k''_6	CheA4/CheA3-P to CheY6 Phosphotransfer	0.775	(µMs) -1
k_7	CheA3-P to CheY6 Reverse phosphotransfer	0.00283	(µMs) -1
k'_7	CheA4/CheA3-P to CheY6 Reverse phosphotransfer	0.00283	(µMs) -1
k''_7	CheA4/CheA3-P to CheY6 Reverse phosphotransfer	0.00283	(µMs) -1
