# Supplementary material for: Split Histidine Kinases Enable Ultrasensitivity and Bistability in Two-Component Signaling Networks
Source: PLoS Comput Biol. 2013 Mar 7;9(3):e1002949. doi: 10.1371/journal.pcbi.1002949 (PMC3591291; doi:10.1371/journal.pcbi.1002949)
Supplement: Table S3 — Parameter values used for the models with additional kinase. (PDF) [file pcbi.1002949.s011.pdf]

Table S3

Parameter	Description	Value	Unit
k_3	Forward rate for phosphorylation complex	1	$(\mu Ms)^{-1}$
k_4	Reverse rate for phosphorylation complex	39	s^{-1}
k^*_3	Forward rate for phosphorylation complex with CheA2	1	$(\mu Ms)^{-1}$
k^*_4	Reverse rate for phosphorylation complex with CheA2	39	s^{-1}
k_5	K_{cat} for phosphorylation of CheA3 by CheA4	varied	s^{-1}
k^*_5	K_{cat} for phosphorylation of CheA2	varied	s^{-1}
k^*_6	CheA2-P to CheY6 Phosphotransfer	0.775	$(\mu Ms)^{-1}$
k^*_7	CheA2-P to CheY6 Reverse phosphotransfer	0.00283	$(\mu Ms)^{-1}$
