# Supplementary material for: Split Histidine Kinases Enable Ultrasensitivity and Bistability in Two-Component Signaling Networks
Source: PLoS Comput Biol. 2013 Mar 7;9(3):e1002949. doi: 10.1371/journal.pcbi.1002949 (PMC3591291; doi:10.1371/journal.pcbi.1002949)
Supplement: Table S4 — Parameter values used for the model of the in vitro experimental system. (PDF) [file pcbi.1002949.s012.pdf]

Table S4

Parameter	Description	Value	Unit
k_1	On rate for binding of CheA3 and CheA4	100	(µMs-^{1})
k_2	Off rate for binding of CheA3 and CheA4	10	s^{-1}
k'_1	On rate for binding of CheA3P1 and CheA4	100	(µMs-^{1})
k''_1	On rate for binding of CheA3P1-P and CheA4	100	(µMs-^{1})
k'_2	Off rate for binding of CheA3-P1 and CheA4	10	s^{-1}
k''_2	Off rate for binding of CheA3P1-P and CheA4	10	s^{-1}
k_6	CheA3-P to CheY6 Phosphotransfer	0.775	(µMs) $^{-1}$
k_7	CheA3-P to CheY6 Reverse phosphotransfer	0.00283	(µMs) $^{-1}$
k'_6	CheA4/CheA3P1-P to CheY6 Phosphotransfer	0.775	(µMs) $^{-1}$
k'_7	CheA4/CheA3P1-P to CheY6 Reverse phosphotransfer	0.00283	(µMs) $^{-1}$
k_8	Autodephosphorylation of CheY6-P	0.169	s^{-1}
k_9	Association of phosphatase assisted dephosphorylation complex	5.6	(µMs) $^{-1}$
k_{10}	Dissociation of phosphatase assisted dephosphorylation complex	0.04	s^{-1}
k_{11}	k_{cat} for phosphatase assisted dephosphorylation	2.5	s^{-1}
$[A3]_{tot}$	Total concentration of CheA3	2.5	µM
$[A4]_{tot}$	Total concentration of CheA4	0,20,40,60	µM
$[Y6]_{tot}$	Total concentration of CheY6	100	µM
[A3P1-P]	Total concentration of CheA3P1-P	30	µM
