# Supplementary material for: Split Histidine Kinases Enable Ultrasensitivity and Bistability in Two-Component Signaling Networks
Source: PLoS Comput Biol. 2013 Mar 7;9(3):e1002949. doi: 10.1371/journal.pcbi.1002949 (PMC3591291; doi:10.1371/journal.pcbi.1002949)
Supplement: Text S1 — Supplementary information on alternative models and their analyses. (PDF) [file pcbi.1002949.s013.pdf]

Split histidine kinases enable ultrasensitivity and bistability in two-component signaling networks

Munia Amin[1, 2] Steven L. Porter[1,*] and Orkun S.Soyer[2,*]

Author Affiliations:
Biosciences, College of Life and Environmental Sciences, University of Exeter, Exeter, UK.
Systems Biology Program, College of Engineering, Computing and Mathematics, University of Exeter, Exeter, UK.

SUPPLEMENTARY INFORMATION

1. **Alternative models considering additional molecules with phosphatase activities.**
2. **Model with an alternative reaction scheme.**
3. **Model with additional kinase, CheA2.**
4. **Analytical solutions for simplified systems with a bifunctional, split kinase vs. Split kinase and stand alone phosphatase.**
5. **Mathematical model for the phosphotransfer experiments.**

1. Models considering additional species with phosphatase activity.

In the basic model describing a split kinase system we have assumed that only free CheA3 has phosphatase activity towards the phosphorylated response regulator CheY6. Here, we relax this assumption by considering additional molecular species with phosphatase activity. We create two alternative models where we separately consider the ability of phosphorylated and complexed CheA3 to act as a phosphatase. These models contain one and two additional reactions respectively, in addition to those reactions considered in the basic model. Below, we list these additional reactions and the resulting ordinary differential equations (ODEs) for each model. Model parameters are given in Table S1 and are mostly derived from the basic model parameters. The effect of having these additional phosphatases on signal-response relationship is shown in Figure S4.

1.1. Model with CheA3-CheA4 and CheA3-CheA4-ATP complexes as phosphatases:

Additional reactions;

$$A3A4 + Y6p \underset{k_{13}}{\overset{k_{12}}{\rightleftarrows}} A3A4Y6p \xrightarrow{k_{14}} A3A4 + Y6 + P_i$$

$$A3A4ATP + Y6p \underset{k_{16}}{\overset{k_{15}}{\rightleftarrows}} A3A4ATPY6p \xrightarrow{k_{17}} A3A4ATP + Y6 + P_i$$

which, combined with the original reactions listed in the main text, results in the following new set of ODEs;

$$\frac{d[A3p]}{dt} = k_5 \cdot [A3A4ATP] + k_7 \cdot [A3] \cdot [Y6p] - k_6 \cdot [A3p] \cdot [Y6]$$

$$\frac{d[A3A4]}{dt} = k_1 \cdot [A3] \cdot [A4] + k_4 \cdot [A3A4ATP] + [A3A4Y6p] \cdot (k_{13} + k_{14}) - [A3A4] \cdot \left(k_2 + k_3 \cdot [ATP] + k_{12} \cdot [Y6p]\right)$$

$$\frac{d[A3A4ATP]}{dt} = k_3 \cdot [A3A4] \cdot [ATP] + [A3A4ATP] \cdot (k_{16} + k_{17}) - [A3A4ATP] \cdot \left(k_4 + k_5 + k_{15} \cdot [Y6p]\right)$$

$$\frac{d[A3Y6p]}{dt} = k_9 \cdot [A3] \cdot [Y6p] - [A3Y6p] \cdot \left(k_{10} + k_{11}\right)$$

$$\frac{d[A3A4Y6p]}{dt} = k_{12} \cdot [Y6p] \cdot [A3A4] - [A3A4Y6p] \cdot (k_{13} + k_{14})$$

$$\frac{d[A3A4ATPY6p]}{dt} = k_{15} \cdot [Y6p] \cdot [A3A4ATP] - [A3A4ATPY6p] \cdot (k_{16} + k_{17})$$

$$\frac{d[Y6p]}{dt} = k_{10} \cdot [A3Y6p] + k_6 \cdot [A3p] \cdot [Y6] + k_{13} \cdot [A3A4Y6p] + k_{16} \cdot [A3A4ATPY6p]$$
$$-[Y6p] \cdot \left(k_7 \cdot [A3] + k_8 + k_9 \cdot [A3] + k_{12} \cdot [A3A4] + k_{15} \cdot [A3A4ATP]\right)$$

1.2. Model with CheA3p as phosphatase:

Additional reactions;

$$A3p + Y6p \underset{k_{19}}{\overset{k_{18}}{\rightleftarrows}} A3pY6p \xrightarrow{k_{20}} A3p + Y6 + P_i$$

which, combined with the original reactions listed in the main text, result in the following new set of ODEs;

$$\frac{d[A3p]}{dt} = k_5 \cdot [A3A4ATP] + k_7 \cdot [A3] \cdot [Y6p] + [A3pY6p] \cdot (k_{19} + k_{20}) - k_6 \cdot [A3p] \cdot [Y6] - k_{18} \cdot [A3p] \cdot [Y6p]$$

$$\frac{d[A3A4]}{dt} = k_1 \cdot [A3] \cdot [A4] + k_4 \cdot [A3A4ATP] - [A3A4] \cdot \left(k_2 + k_3 \cdot [ATP]\right)$$

$$\frac{d[A3A4ATP]}{dt} = k_3 \cdot [A3A4] \cdot [ATP] - [A3A4ATP] \cdot \left(k_4 + k_5\right)$$

$$\frac{d[A3Y6p]}{dt} = k_9 \cdot [A3] \cdot [Y6p] - [A3Y6p] \cdot \left(k_{10} + k_{11}\right)$$

$$\frac{d[A3pY6p]}{dt} = k_{18} \cdot [A3p] \cdot [Y6p] - [A3pY6p] \cdot \left(k_{19} + k_{20}\right)$$

$$\frac{d[Y6p]}{dt} = k_{10} \cdot [A3Y6p] + k_6 \cdot [A3p] \cdot [Y6] + k_{19} \cdot [A3pY6p] - [Y6p] \cdot \left(k_7 \cdot [A3] + k_8 + k_9 \cdot [A3] + k_{18} \cdot [A3p]\right)$$

2. Model with an alternative reaction scheme.

In the basic model describing a split kinase system and discussed in the main text, we have assumed that the phosphorylation of the CheA3 by CheA4 results in the dissociation of the CheA3:CheA4 complex. Here, we relaxed this assumption to create an alternative model. In this model, we allowed for the possibility that phosphorylated CheA3 remains in complex with CheA4 and that this CheA3p:CheA4 complex is also capable of acting as phosphatase towards CheY6p (corresponding reaction rates k'_5, k'_6 and k''_6). We find that having these reactions in the model does not affect the level of ultrasensitivity but can lead to loss of bistability (Figure S6). Note, that besides these reactions, this alternative model is the same as the basic model and only considers phosphatase activity by free CheA3. Model parameters are given in Table S2 and are mostly derived from the basic model parameters. As in the basic model (Figure 2D and S4), considering alternative phosphatases in this alternative model significantly reduces ultrasensitivity and leads to loss of bistability (data not shown).

This alternative model contains the following reactions;

$$A3 + A4 \underset{k_2}{\overset{k_1}{\rightleftarrows}} A3A4$$
$$A3A4 + ATP \underset{k_4}{\overset{k_3}{\rightleftarrows}} A3A4ATP \xrightarrow{k_5} A3p + A4 + ADP$$
$$A3p + A4 \underset{k'_2}{\overset{k'_1}{\rightleftarrows}} A3pA4$$
$$A3A4ATP \xrightarrow{k'_5} A3pA4 + ADP$$
$$A3p + Y6 \underset{k_7}{\overset{k_6}{\rightleftarrows}} A3 + Y6p$$
$$A3pA4 + Y6 \underset{k'_7}{\overset{k'_6}{\rightleftarrows}} A3A4 + Y6p$$
$$A3pA4 + Y6 \underset{k''_7}{\overset{k''_6}{\rightleftarrows}} A3 + A4 + Y6p$$
$$Y6p \xrightarrow{k_8} Y6 + P_i$$
$$A3 + Y6p \underset{k_{10}}{\overset{k_9}{\rightleftarrows}} A3Y6p \xrightarrow{k_{11}} A3 + Y6 + P_i$$

resulting in the following set of ODEs;

$$\frac{d[A3A4]}{dt} = [A3]\cdot[A4]\cdot k_1 - [A3A4]\cdot k_2 + [A3A4ATP]\cdot k_4 - [A3A4]\cdot[ATP]\cdot k_3$$
$$+[A3pA4]\cdot[Y6]\cdot k'_6 - [A3A4]\cdot[Y6p]\cdot k'_7$$
$$\frac{d[A3A4ATP]}{dt} = [A3A4]\cdot[ATP]\cdot k_3 - [A3A4ATP]\cdot(k_4 + k_5 + k'_5)$$
$$\frac{d[A3p]}{dt} = [A3A4ATP]\cdot k_5 + [Y6p]\cdot[A3]\cdot k_7 - [A3p]\cdot([Y6]\cdot k_6 + [A4]\cdot k'_1)$$
$$+[A3pA4]\cdot k'_2$$
$$\frac{d[A3pA4]}{dt} = [A3p]\cdot[A4]\cdot k'_1 - [A3pA4]\cdot(k'_2 + [Y6]\cdot k'_6 + [Y6]\cdot k''_6) + [A3A4ATP]\cdot k'_5$$
$$+[A3A4]\cdot[Y6p]\cdot k'_7 + [A3]\cdot[A4]\cdot[Y6p]\cdot k''_7$$
$$\frac{d[A3Y6p]}{dt} = [A3]\cdot[Y6p]\cdot k_9 - [A3Y6p]\cdot(k_{10} + k_{11})$$
$$\frac{d[Y6p]}{dt} = [A3p]\cdot[Y6]\cdot k_6 - [Y6p]\cdot[A3]\cdot k_7 - [A3A4]\cdot[Y6p]\cdot k'_7 + [A3pA4]\cdot([Y6]\cdot k'_6 + [Y6]\cdot k''_6)$$
$$-[A3]\cdot[A4]\cdot[Y6p]\cdot k''_7 + [A3Y6p]\cdot k_{10} - [A3]\cdot[Y6p]\cdot k_9 - [Y6p]\cdot k_8$$

3. Model with additional kinase, CheA2

In the basic model describing a split kinase system we have only considered phosphorylation of the response regulator (i.e. CheY6) by the split kinase. *In vivo*, cross-talk from other kinases could also result in the phosphorylation of the response regulator. For example, in *Rhodobacter sphaeroides,* another kinase, CheA2 is known to phosphorylate CheY6 [27]. Here, we determine the effect of having such an additional kinase on the response dynamics generated by the split kinase. We created a model having this additional

kinase activity and analysed the signal-response relationship in the system under a range of phosphotransfer rates from such an additional kinase (Figure S7). Model parameters are given in Table S3 and are mostly derived from the basic model parameters. This model contains two additional reactions:

$$A2 + ATP \underset{k^{*}_4}{\overset{k^{*}_3}{\rightleftarrows}} A2 \cdot ATP \xrightarrow{k^{*}_5} A2p$$
$$A2p + Y6 \underset{k^{*}_7}{\overset{k^{*}_6}{\rightleftarrows}} Y6p + A2$$

which, combined with the original reactions listed in the main text, results in the following new set of ODEs;

$$\frac{d[A3A4]}{dt} = A3 \cdot A4 \cdot k_1 - A3A4 \cdot k_2 + A3A4ATP \cdot k_4 - A3A4 \cdot ATP \cdot k_3$$
$$\frac{d[A3A4ATP]}{dt} = A3A4 \cdot ATP \cdot k_3 - A3A4ATP \cdot (k_4 + k_5)$$
$$\frac{d[A3p]}{dt} = A3A4ATP \cdot k_5 + Y6p \cdot A3 \cdot k_7 - A3p \cdot Y6 \cdot k_6$$
$$\frac{d[A2p]}{dt} = A2ATP \cdot k^{*}_5 + Y6p \cdot A2 \cdot k^{*}_7 - A2p \cdot Y6 \cdot k^{*}_6$$
$$\frac{d[A2ATP]}{dt} = A2 \cdot ATP \cdot k^{*}_3 - A2ATP \cdot (k^{*}_4 + k^{*}_5)$$
$$\frac{d[A3Y6p]}{dt} = Y6p \cdot A3 \cdot k_9 - A3Y6p \cdot (k_{10} + k_{11})$$
$$\frac{d[Y6p]}{dt} = A3p \cdot Y6 \cdot k_6 - Y6p \cdot A3 \cdot k_7 + A3Y6p \cdot k_{10} - Y6p \cdot A3 \cdot k_9$$
$$+ A2p \cdot Y6 \cdot k^{*}_6 + Y6p \cdot A2 \cdot k^{*}_7 - Y6p \cdot k_8$$

4. Analytical solutions for simplified systems with a bifunctional, split kinase vs. split kinase with a stand-alone phosphatase.

Besides using the chemical reaction network theory to analse different models (see discussion in the main text), we have also derived analytical solutions for a simplified reaction scheme for a bifunctional split kinase and also for a monofunctional split kinase with a stand-alone phosphatase (i.e. where dephosphorylation of the response regulator is mediated by a separate phosphatase).

4.1. Simplified reaction scheme and analytical solution for a system with bifunctional, split kinase.

In this simplified scheme, we assume that all phosphotransfer and dephosphorylation reactions occur very fast and that complex formation can be ignored. The reaction scheme we consider is;

$$A3 + A4 \underset{k_2}{\overset{k_1}{\rightleftarrows}} A3A4$$
$$A3A4 + ATP \xrightarrow{k_5} A3p + A4 + ADP$$
$$A3p + Y6 \xrightarrow{k_6} A3 + Y6p$$
$$Y6p + A3 \xrightarrow{k_9} Y6 + A3 + P_i$$
$$Y6p \xrightarrow{k_8} Y6 + P_i$$

which results in the following ODEs;

$$\frac{d[A3A4]}{dt} = [A3]\cdot[A4]\cdot k_1 - [A3A4]\cdot(k_2 + k_5)$$
$$\frac{d[A3p]}{dt} = [A3A4]\cdot k_5 - [A3p]\cdot[Y6]\cdot k_6$$
$$\frac{d[Y6p]}{dt} = [A3p]\cdot[Y6]\cdot k_6 - [Y6p]\cdot[A3]\cdot k_9 - [Y6p]\cdot k_8$$

We first define the conservation relations in the system:

$$[A3]_{tot} = [A3] + [A3p] + [A3A4]$$
$$[A4]_{tot} = [A4] + [A3A4]$$
$$[Y6]_{tot} = [Y6] + [Y6p]$$

At steady state, all of the above ODEs would be equal to zero, allowing us to derive the steady state expression for phosphorylated CheY6. Following simple algebra, we arrive at a quartic equation;

$$A3\cdot A4\cdot k_1 - A3A4\cdot(k_2 + k_5) = 0 \qquad (1)$$

$$A3A4\cdot k_5 - A3p\cdot Y6\cdot k_6 = 0 \qquad (2)$$

$$A3p\cdot Y6\cdot k_6 - Y6p\cdot A3\cdot k_9 - Y6p\cdot k_8 = 0 \qquad (3)$$

Solving equation 2 and 3, we get

$$A3p\cdot Y6\cdot k_6 = Y6p\cdot A3\cdot k_9 + Y6p\cdot k_8$$
$$Y6p\cdot(A3_{tot} - A3p - A3A4)\cdot k_9 + Y6p\cdot k_8 = A3p\cdot Y6\cdot k_6$$

$$A3p \cdot Y6 \cdot k_6 = A3A4 \cdot k_5$$

$$A3p = \frac{A3A4 \cdot k_5}{Y6 \cdot k_6}$$

$$Y6p \cdot (A3_{tot} - \frac{A3A4 \cdot k_5}{Y6 \cdot k_6} - A3A4) \cdot k_9 + Y6p \cdot k_8 = A3A4 \cdot k_5$$

$$\frac{Y6p \cdot A3_{tot} \cdot Y6 \cdot k_6 \cdot k_9 - Y6p \cdot A3A4 \cdot k_5 \cdot k_9 - Y6p \cdot Y6 \cdot A3A4 \cdot k_6 \cdot k_9 + Y6p \cdot Y6 \cdot k_8 \cdot k_6}{Y6 \cdot k_6} = A3A4 \cdot k_5$$

$$Y6p \cdot A3_{tot} \cdot Y6 \cdot k_6 \cdot k_9 - Y6p \cdot A3A4 \cdot k_5 \cdot k_9 - Y6p \cdot Y6 \cdot A3A4 \cdot k_6 \cdot k_9 + Y6p \cdot Y6 \cdot k_8 \cdot k_6 = A3A4 \cdot Y6 \cdot k_5 \cdot k_6$$

$$A3A4 \cdot (Y6p \cdot k_5 \cdot k_9 + Y6p \cdot Y6 \cdot k_6 \cdot k_9 + Y6 \cdot k_5 \cdot k_6) = Y6p \cdot A3_{tot} \cdot Y6 \cdot k_6 \cdot k_9 + Y6p \cdot Y6 \cdot k_8 \cdot k_6$$

$$A3A4 = \frac{Y6p \cdot A3_{tot} \cdot Y6 \cdot k_6 \cdot k_9 + Y6p \cdot Y6 \cdot k_8 \cdot k_6}{Y6p \cdot k_5 \cdot k_9 + Y6p \cdot Y6 \cdot k_6 \cdot k_9 + Y6 \cdot k_5 \cdot k_6}$$

$$A3A4 = \frac{Y6p \cdot A3_{tot} \cdot Y6 \cdot k_6 \cdot k_9 + Y6p \cdot Y6 \cdot k_8 \cdot k_6}{N} [N = Y6p \cdot k_5 \cdot k_9 + Y6p \cdot Y6 \cdot k_6 \cdot k_9 + Y6 \cdot k_5 \cdot k_6]$$

(4)

$$A3p = (\frac{Y6p \cdot A3_{tot} \cdot Y6 \cdot k_6 \cdot k_9 + Y6p \cdot Y6 \cdot k_8 \cdot k_6}{Y6p \cdot k_5 \cdot k_9 + Y6p \cdot Y6 \cdot k_6 \cdot k_9 + Y6 \cdot k_5 \cdot k_6}) \cdot \frac{k_5}{Y6 \cdot k_6}$$

$$A3p = \frac{Y6p \cdot A3_{tot} \cdot k_5 \cdot k_6 \cdot k_9 + Y6p \cdot k_5 \cdot k_8 \cdot k_6}{N \cdot k_6} \qquad (5)$$

From equation 1,

$$A3 \cdot A4 \cdot k_1 = A3A4 \cdot (k_2 + k_5)$$

$$A3 \cdot A4 = A3A4 \cdot \frac{(k_2 + k_5)}{k_1}$$

$$(A3_{tot} - A3p - A3A4) \cdot (A4_{tot} - A3A4) = A3A4 \cdot a[a = \frac{(k_2 + k_5)}{k_1}]$$

$$A3_{tot} \cdot A4_{tot} - A3p \cdot A4_{tot} - A3A4 \cdot (A4_{tot} + A3_{tot} + a) + A3p \cdot A3A4 + A3A4^2 = 0$$

(6)

Putting value of A3p and A3A4 from eq 4&5 into 6, we get

$$A3_{tot}\cdot A4_{tot} - \left(\frac{Y6p\cdot A3_{tot}\cdot k_5\cdot k_6\cdot k_9 + Y6p\cdot k_5\cdot k_8\cdot k_6}{N\cdot k_6}\right)\cdot A4_{tot} - \left(\frac{Y6p\cdot A3_{tot}\cdot Y6\cdot k_6\cdot k_9 + Y6p\cdot Y6\cdot k_8\cdot k_6}{N}\right)\cdot (A4_{tot} + A3_{tot} + a)$$
$$+\left(\frac{Y6p\cdot A3_{tot}\cdot k_5\cdot k_6\cdot k_9 + Y6p\cdot k_5\cdot k_8\cdot k_6}{N\cdot k_6}\right)\cdot\left(\frac{Y6p\cdot A3_{tot}\cdot Y6\cdot k_6\cdot k_9 + Y6p\cdot Y6\cdot k_8\cdot k_6}{N}\right) + \left(\frac{Y6p\cdot A3_{tot}\cdot Y6\cdot k_6\cdot k_9 + Y6p\cdot Y6\cdot k_8\cdot k_6}{N}\right)^2 = 0$$

$$\frac{\begin{array}{l} A3_{tot}\cdot A4_{tot}\cdot N^2\cdot k_6 - N\cdot Y6p\cdot c - N\cdot k_6\cdot (A4_{tot} + A3_{tot} + a)\cdot (A3_{tot}\cdot k_6\cdot k_9 + k_8\cdot k_6)\cdot Y6p\cdot Y6 \\ +(A3_{tot}\cdot k_5\cdot k_6\cdot k_9 + k_5\cdot k_8\cdot k_6)\cdot Y6p\cdot (Y6p\cdot A3_{tot}\cdot Y6\cdot k_6\cdot k_9 + Y6p\cdot Y6\cdot k_8\cdot k_6) + k_6\cdot (A3_{tot}\cdot k_6\cdot k_9 + k_8\cdot k_6)^2\cdot (Y6p\cdot Y6)^2 \end{array}}{N^2\cdot k_6} = 0$$

$$\begin{array}{l} b\cdot N^2 - N\cdot Y6p\cdot c - N\cdot d\cdot Y6p\cdot Y6 \\ +e\cdot Y6p\cdot (Y6p\cdot A3_{tot}\cdot Y6\cdot k_6\cdot k_9 + Y6p\cdot Y6\cdot k_8\cdot k_6) + f\cdot (Y6p\cdot Y6)^2 = 0 \end{array}$$

Where following were given by,

$$\begin{array}{l} [b = A3_{tot}\cdot A4_{tot}\cdot k_6, \\ c = A4_{tot}\cdot (A3_{tot}\cdot k_5\cdot k_6\cdot k_9 + k_5\cdot k_8\cdot k_6), \\ d = (A4_{tot} + A3_{tot} + a)\cdot k_6\cdot (A3_{tot}\cdot k_6\cdot k_9 + k_8\cdot k_6), \\ e = (A3_{tot}\cdot k_5\cdot k_6\cdot k_9 + k_5\cdot k_8\cdot k_6), \\ f = k_6\cdot (A3_{tot}\cdot k_6\cdot k_9 + k_8\cdot k_6)^2] \end{array}$$

Putting back the value of N and solving it we get,

$$\begin{array}{l} Y6p^4\cdot (k_6^2\cdot k_9^2\cdot b - k_6\cdot k_9\cdot d + f) \\ +Y6p^3\cdot (2\cdot k_5\cdot k_6^2\cdot k_9\cdot b - 2\cdot k_5\cdot k_6\cdot k_9^2\cdot b - 2\cdot Y6_{tot}\cdot k_6^2\cdot k_9^2\cdot b + k_6\cdot k_9\cdot c - k_5\cdot k_6\cdot d + 2\cdot Y6_{tot}\cdot k_6\cdot k_9\cdot d \\ +k_5\cdot k_9\cdot d - A3_{tot}\cdot k_6\cdot k_9\cdot e - k_6\cdot k_8\cdot e - 2\cdot Y6_{tot}\cdot f) \\ +Y6p^2\cdot (k_5^2\cdot k_9^2\cdot b + Y6_{tot}^2\cdot k_6^2\cdot k_9^2\cdot b + k_5^2\cdot k_6^2\cdot b + 2\cdot Y6_{tot}\cdot k_5\cdot k_6\cdot k_9^2\cdot b - 4\cdot Y6_{tot}\cdot k_5\cdot k_6^2\cdot k_9\cdot b \\ -2\cdot k_5^2\cdot k_6\cdot k_9\cdot b - k_5\cdot k_9\cdot c - Y6_{tot}\cdot k_6\cdot k_9\cdot c + k_5\cdot k_6\cdot c - Y6_{tot}\cdot k_5\cdot k_9\cdot d - Y6_{tot}^2\cdot k_6\cdot k_9\cdot d \\ +2\cdot Y6_{tot}\cdot k_5\cdot k_6\cdot d + A3_{tot}\cdot Y6_{tot}\cdot k_6\cdot k_9\cdot e + Y6_{tot}\cdot k_6\cdot k_8\cdot e + Y6_{tot}^2\cdot f) \\ +Y6p\cdot (2\cdot Y6_{tot}^2\cdot k_5\cdot k_6^2\cdot k_9\cdot b - 2\cdot Y6_{tot}\cdot k_5^2\cdot k_6^2\cdot b + 2\cdot Y6_{tot}\cdot k_5^2\cdot k_6\cdot k_9\cdot b - Y6_{tot}\cdot k_5\cdot k_6\cdot c - Y6_{tot}^2\cdot k_5\cdot k_6\cdot d) \\ +Y6_{tot}^2\cdot k_5^2\cdot k_6^2\cdot b = 0 \end{array}$$

The emergence of the quartic expression for the steady state level of phosphorylated CheY6 indicates the potential of this system to reach bistability and high level of nonlinearity even without considering complex formation. To confirm bistability, we have analysed the model shown above and a similar one (see Supplementary Text S2) using chemical reaction network theory. This confirmed the potential of bistability in both of these models (see also discussion in the main text). Furthermore, we have analysed the above simplified model by evaluating the analytical solution over the same signal range as for the basic model. For reactions that were modeled as bi- or uni-molecular both in the basic model and this simplified model, we have used the parameters as in the basic model. For reactions that were modeled via complex formation in the basic model (e.g. the A3p mediated dephosphorylation of Y6p), we have explored different parameter values. In line with the results of the chemical reaction network theory, this analysis

confirmed that the modeled system displays bistability (i.e. multiple permissible steady states) in a biologically permissible parameter regime.

4.2. Simplified reaction scheme and analytical solution for a system with a monofunctional, split kinase and stand-alone phosphatase.

As before, we assume that all phosphotransfer and dephosphorylation reactions occur very fast and ignore the formation of complexes. The reaction scheme we consider is;

$$A3 + A4 \underset{k_2}{\overset{k_1}{\rightleftarrows}} A3A4$$
$$A3A4 + ATP \xrightarrow{k_5} A3p + A4 + ADP$$
$$A3p + Y6 \xrightarrow{k_6} A3 + Y6p$$
$$Y6p + X \xrightarrow{k_9} Y6 + X + P_i$$
$$Y6p \xrightarrow{k_8} Y6 + P_i$$

We first define the conservation relations in the system:

$$[A3]_{tot} = [A3] + [A3p] + [A3A4]$$
$$[A4]_{tot} = [A4] + [A3A4]$$
$$[Y6]_{tot} = [Y6p] + [Y6]$$
$$[X]_{tot} = [X]$$

which results in the following ODEs;

$$\frac{d[A3A4]}{dt} = [A3]\cdot[A4]\cdot k_1 - [A3A4]\cdot(k_2 + k_5)$$
$$\frac{d[A3p]}{dt} = [A3A4]\cdot k_5 - [A3p]\cdot[Y6]\cdot k_6$$
$$\frac{d[Y6p]}{dt} = [A3p]\cdot[Y6]\cdot k_6 - [Y6p]\cdot[X]\cdot k_9 - [Y6p]\cdot k_8$$

At steady state, all of the above ODEs would be equal to zero, allowing us to derive the steady state expression for phosphorylated CheY6. Following simple algebra, we arrive at a cubic equation;

$$A3\cdot A4\cdot k_1 - A3A4\cdot(k_2 + k_5) = 0 \qquad (1)$$

$$A3A4\cdot k_5 - A3p\cdot Y6\cdot k_6 = 0 \qquad (2)$$

$$A3p\cdot Y6\cdot k_6 - Y6p\cdot X\cdot k_9 - Y6p\cdot k_8 = 0 \qquad (3)$$

Solving equation 2 and 3, we got

$$Y6p = \frac{A3A4 \cdot k_5}{X_{tot} \cdot k_9 + k_8}$$
$$Y6p = A3A4 \cdot a$$
$$A3A4 = \frac{Y6p}{a}$$

Where a is given by; $a = \frac{k_5}{X_{tot} \cdot k_9 + k_8}$

From equation 2,

$$A3p = \frac{Y6p \cdot c}{Y6}$$

Where c is given by; $c = \frac{k_5}{k_6 \cdot a}$

From equation 1, we get

$$A3A4 \cdot b = (A3_{tot} - A3p - A3A4) \cdot (A4_{tot} - A3A4) \qquad (4)$$

Putting A3A4 and A3p values in equation 4, we get

$$Y6p^3 \cdot Y6_{tot} + Y6p^2 \cdot (-A3_{tot} \cdot a - b \cdot a - c \cdot a - A4_{tot} \cdot a - Y6_{tot}) +$$
$$Y6p \cdot (b \cdot a \cdot Y6_{tot} + A4_{tot} \cdot c \cdot a^2 + A3_{tot} \cdot A4_{tot} \cdot a^2 +$$
$$Y6_{tot} \cdot A4_{tot} \cdot a + A3_{tot} \cdot a \cdot Y6_{tot)} - A3_{tot} \cdot Y6_{tot} \cdot A4_{tot} \cdot a^2 = 0$$

Where b is given by; $b = \frac{(k_2 + k_5)}{k_1}$

The emergence of the cubic expression for the steady state level of phosphorylated CheY6 indicates less nonlinearity in the system compared with the system with a bifunctional split kinase (previous section). A numerical analysis using this analytical expression (as done in the previous section), shows that in the similar parameter ranges where the previous model shows bistability, this one does not. Again, this is inline and as expected from the results of the chemical reaction network theory, which shows no possibility of bistability in this model (see main text and Supplementary Text S3).

5. Mathematical model of the phosphotransfer experiments

We developed a mathematical model of the specific in vitro experimental setup used to test whether CheA4 can inhibit the phosphatase activity of CheA3. In particular, these experiments employed a truncated form of CheA3, CheA3P1, that lacks phosphatase activity and that can be isolated in a fully phosphorylated form (7). We mixed CheA3P1-P with CheY6 in the absence of ATP and monitored phosphotransfer to CheY6 and its subsequent dephosphorylation by CheA3. In the model, CheA3P1-P was assumed to have the same phosphotransfer kinetics as CheA3. We also assumed that CheA3P1 and CheA3P1-P can bind to CheA4 at the same rate as CheA3. The resulting set of reactions in the system are;

$$A3 + A4 \underset{k_2}{\overset{k_1}{\rightleftarrows}} A3A4$$

$$P1 + A4 \underset{k'_2}{\overset{k'_1}{\rightleftarrows}} A4P1$$

$$P1p + A4 \underset{k''_2}{\overset{k''_1}{\rightleftarrows}} A4P1p$$

$$A3p + Y6 \underset{k_7}{\overset{k_6}{\rightleftarrows}} A3 + Y6p$$

$$A4P1p + Y6 \underset{k`_7}{\overset{k`_6}{\rightleftarrows}} A4P1 + Y6p$$

$$Y6p \xrightarrow{k_8} Y6 + P_i$$

$$A3 + Y6p \underset{k_{10}}{\overset{k_9}{\rightleftarrows}} A3Y6p \xrightarrow{k_{11}} A3 + Y6 + P_i$$

$$A3A4 + Y6p \underset{k_{13}}{\overset{k_{12}}{\rightleftarrows}} A3A4Y6p \xrightarrow{k_{14}} A3A4 + Y6 + P_i$$

giving rise to the following ODEs;

$$\frac{d[A3A4]}{dt} = A3 \cdot A4 \cdot k_1 - A3A4 \cdot k_2 - A3A4 \cdot Y6p \cdot k_{12} + A3A4Y6p \cdot (k_{13} + k_{14})$$

$$\frac{d[A4P1]}{dt} = P1 \cdot A4 \cdot k'_1 - A4P1 \cdot k'_2 + A4P1p \cdot Y6 \cdot k`_6 - A4P1 \cdot Y6p \cdot k`_7$$

$$\frac{d[P1]}{dt} = A4P1 \cdot k'_2 - P1 \cdot A4 \cdot k'_1 + P1p \cdot Y6 \cdot k_6 - P1 \cdot Y6p \cdot k_7$$

$$\frac{d[A4P1p]}{dt} = A4 \cdot P1p \cdot k''_2 - A4P1p \cdot k''_1 - A4P1p \cdot Y6 \cdot k`_6 + A4P1 \cdot Y6p \cdot k`_7$$

$$\frac{d[A3Y6p]}{dt} = A3 \cdot Y6p \cdot k_9 - A3Y6p \cdot (k_{10} + k_{11})$$

$$\frac{d[A3A4Y6p]}{dt} = A3A4 \cdot Y6p \cdot k_{12} - A3A4Y6p \cdot (k_{13} + k_{14})$$

$$\frac{d[Y6p]}{dt} = P1p \cdot Y6 \cdot k_6 - P1 \cdot Y6p \cdot k_7 + A4P1p \cdot Y6 \cdot k`_6 - A4P1 \cdot Y6p \cdot k`_7 + A3Y6p \cdot k_{10} - A3 \cdot Y6p \cdot k_9$$
$$+ A3A4Y6p \cdot k_{13} - A3A4 \cdot Y6p \cdot k_{12} - Y6p \cdot k_8$$

We numerically solved this system using parameter values given in Supplemental Table 4 and in the presence of different levels of CheA4. By fitting a first-order exponential decay curve to this simulation data, we estimated the half-time of phosphorylated CheY6 (k_{obs}) shown in Figure 4. Under the assumption that CheA4 and CheA3:CheA4 complex are not capable of CheY6-P dephosphorylation, this model predicts that increasing CheA4 levels would slow the CheY6-P dephosphorylation kinetics by sequestering free CheA3. We found that this model provides a good qualitative match to the experimental observations (Figure 4 in the main text).
