# Supplementary material for: Split Histidine Kinases Enable Ultrasensitivity and Bistability in Two-Component Signaling Networks
Source: PLoS Comput Biol. 2013 Mar 7;9(3):e1002949. doi: 10.1371/journal.pcbi.1002949 (PMC3591291; doi:10.1371/journal.pcbi.1002949)
Supplement: Text S2 — Results of the analytical analysis of the basic model. The file contains the reaction system considered and the report produced with the Chemical Network Tool v2.2 (http://www.chbmeng.ohio-state.edu/~feinberg/crntwin/). (DOC) [file pcbi.1002949.s014.doc]

=====================

BASIC REPORT

=====================

Reaction network:

¯¯¯¯¯¯¯¯¯¯¯¯¯¯¯¯

A + B <-> AB

AB <-> Ap + B

Ap + Y <-> Yp + A

Yp + A <-> YpA

YpA -> Y + A

Yp -> Y

Remark: None.

¯¯¯¯¯¯

Graphical Properties

====================

Number of complexes = 9

Number of linkage classes = 3:

Linkage class no. 1: {A + B, AB, Ap + B}

Linkage class no. 2: {Ap + Y, Yp + A, YpA, Y + A}

Linkage class no. 3: {Yp, Y}

Number of TERMINAL strong linkage classes = 3:

Strong linkage class no. 1: {A + B, AB, Ap + B}

Strong linkage class no. 2: {Y + A}

Strong linkage class no. 3: {Y}

Number of NON-TERMINAL strong linkage classes = 2:

Strong linkage class no. 4: {Ap + Y, Yp + A, YpA}

Strong linkage class no. 5: {Yp}

The network is neither reversible nor weakly reversible.

Rank Information

================

Rank of entire network = 4

Deficiency Information

======================

Deficiency of entire network = 2

Deficiency of linkage class no. 1 = 0

Deficiency of linkage class no. 2 = 0

Deficiency of linkage class no. 3 = 0

Analysis

========

This is a deficiency two network. It is an excellent candidate for application

of HIGHER DEFICIENCY THEORY (tailored mostly to networks with deficien-

cies greater than one).

Whether results will be obtained, will depend on whether or not the reaction

network has certain additional structural attributes that help reduce the problem

to a study of systems of linear inequalities.

If a network is "good", higher deficiency theory will determine, either

affirmatively or negatively, whether there are positive rate constant values

such that the corresponding mass action differential equations admit multiple

(positive) steady states. If the answer is affirmative, higher deficiency

theory will generate a sample set of rate constants and a pair of distinct

steady states that are consistent with those rate constants.

If a network is "bad", some additional nonlinear analysis might be required,

and the program might not be able to ascertain the network's capacity for

multiple positive steady states. If definite conclusions can be reached they

they will be reported. Otherwise the program will tell you that it cannot reach

a conclusion.

Higher deficiency theory will also determine, either affirmatively or

negatively, whether there can exist a set of rate constants such that the

corresponding mass action differential equations admit a positive steady

state having a zero eigenvalue (corresponding to an eigenvector in the

stoichiometric subspace). When the answer is affirmative, the theory will

produce such a set of rate constants, a positive steady state, and an

eigenvector (in the stoichiometric subspace) corresponding to an eigenvalue

of zero. Results of this kind are contained after running the Zero Eigenvalue

Report.

HIGHER DEFICIENCY REPORT: NoName1

=================================

Analysis

========

Taken with mass action kinetics, the network DOES have the capacity for

multiple steady states. That is, there are rate constants that give rise to

two or more positive (stoichiometrically compatible) steady states --

you'll see an example below -- and also rate constants for which there is a

steady state having an eigenvector (in the stoichiometric subspace)

corresponding to an eigenvalue of zero. (To construct rate constants that

give a degenerate steady state, use the Zero Eigenvalue Report.)

A mass action system example is also given below:

Example No. 1: Multiple Steady States

¯¯¯¯¯¯¯¯¯¯¯¯¯¯¯¯¯¯¯¯¯¯¯¯¯¯¯¯¯¯¯¯¯¯¯¯¯

The following mass action system gives rise to multiple steady states:

A + B ---29654.369-> AB

AB ---606.38599-> A + B

AB ---1206.1729-> Ap + B

Ap + Y ---1519.9712-> Yp + A

Yp + A -------1-----> Ap + Y

Yp + A ---1149.1451-> YpA

YpA ---5.9882822-> Yp + A

YpA ---6875.4169-> Y + A

Yp ---25.523566-> Y

Ap + B ---19.407423-> AB

The steady states shown below are both consistent with the mass

action system indicated.

Steady State No. 1 Species Steady State No. 2

¯¯¯¯¯¯¯¯¯¯¯¯¯¯¯¯¯¯ ¯¯¯¯¯¯¯ ¯¯¯¯¯¯¯¯¯¯¯¯¯¯¯¯¯¯

5.1796786 A 1.2839 E-2

0.35150954 B 19.19177

29.804854 AB 10.964593

5.1668395 ATP 5.1668395

1 ABATP 1

1 AATP 1

4.5662364 Ap 33.740176

5.1796786 Y 1.2839 E-2

6.0139605 Yp 16.347639

1 AYp 1

1 ApB 1

5.2018896 YpA 3.5050 E-2

Eigenvalues for Steady State No. 1

¯¯¯¯¯¯¯¯¯¯¯¯¯¯¯¯¯¯¯¯¯¯¯¯¯¯¯¯¯¯¯¯¯¯

-5339.4431

-3.2062592

(-547.36202) - i (187.88484)

(-547.36202) + i (187.88484)

Steady State No. 1 is asymptotically stable.

Eigenvalues for Steady State No. 2

¯¯¯¯¯¯¯¯¯¯¯¯¯¯¯¯¯¯¯¯¯¯¯¯¯¯¯¯¯¯¯¯¯¯

-18910.947

-209.5346

-69.4424

-1656.3006

Steady State No. 2 is asymptotically stable.

References

¯¯¯¯¯¯¯¯¯¯

1. Feinberg, M., Chemical reaction network structure and the stability of comp-

lex isothermal reactors. I. The deficiency zero and deficiency one theorems,

Chem. Eng. Science, 42, 2229-2268 (1987).

2. Ellison, P. and Feinberg, M. How catalytic mechanisms reveal themselves

in multiple steady state data. I. Basic principles, The Journal of Molecular

Catalysis A: Chemical, 154, 155 - 167, 2000.

3. Ellison, P. PhD. Thesis. Rochester, NY: Department of Chemical Engineer-

ing, University of Rochester; 1998. The advanced deficiency algorithm

and itsapplications to mechanism discrimination.

4. Ji, H. PhD. Thesis. Columbus, OH: Department of Mathematics, The Ohio

State University; 2011. Uniqueness of equilibria for complex chemical reaction

networks.
