# Supplementary material for: Split Histidine Kinases Enable Ultrasensitivity and Bistability in Two-Component Signaling Networks
Source: PLoS Comput Biol. 2013 Mar 7;9(3):e1002949. doi: 10.1371/journal.pcbi.1002949 (PMC3591291; doi:10.1371/journal.pcbi.1002949)
Supplement: Text S4 — Results of the analytical analysis of a model with a monofunctional kinase. The file contains the reaction system considered and the report produced with the Chemical Network Tool v2.2 (http://www.chbmeng.ohio-state.edu/~feinberg/crntwin/). (DOC) [file pcbi.1002949.s016.doc]

=====================

BASIC REPORT: NoName1

=====================

Reaction network:

¯¯¯¯¯¯¯¯¯¯¯¯¯¯¯¯

Ap + Y <-> Yp + A

Yp + A <-> YpA

Yp -> Y

A <-> Ap

Remark: None.

¯¯¯¯¯¯

Graphical Properties

====================

Number of complexes = 7

Number of linkage classes = 3:

Linkage class no. 1: {Ap + Y, Yp + A, YpA}

Linkage class no. 2: {Yp, Y}

Linkage class no. 3: {A, Ap}

Number of TERMINAL strong linkage classes = 3:

Strong linkage class no. 1: {Ap + Y, Yp + A, YpA}

Strong linkage class no. 2: {A, Ap}

Strong linkage class no. 3: {Y}

Number of NON-TERMINAL strong linkage classes = 1:

Strong linkage class no. 4: {Yp}

The network is neither reversible nor weakly reversible.

Rank Information

================

Rank of entire network = 3

Deficiency Information

======================

Deficiency of entire network = 1

Deficiency of linkage class no. 1 = 0

Deficiency of linkage class no. 2 = 0

Deficiency of linkage class no. 3 = 0

Analysis

========

This is a regular deficiency one network. It is an excellent candi-

date for application of DEFICIENCY ONE THEORY.

Deficiency one theory will determine, either affirmatively or nega-

tively, whether there are positive rate constant values such that the corre-

sponding mass action differential equations admit multiple (positive) steady

states. If the answer is affirmative, deficiency one theory will generate a

sample set of rate constants and a pair of distinct steady states that are

consistent with those rate constants. To get this informatoin, you should

run the Deficiency One Report.

Deficiency one theory will also determine, either affirmatively or

negatively, whether there can exist a set of rate constants such that the

corresponding mass action differential equations admit a positive steady

state having a zero eigenvalue (corresponding to an eigenvector in the

stoichiometric subspace). When the answer is affirmative, the theory will

produce such a set of rate constants, a positive steady state, and an

eigenvector (in the stoichiometric subspace) corresponding to an eigenvalue

of zero. To get this information, run the Zero Eigenvalue Report (after

running the Deficiency Zero Report).

==============================

DEFICIENCY ONE REPORT: NoName1

==============================

Analysis

========

Taken with mass action kinetics, the network CANNOT admit multiple posi-

tive steady states or a degenerate positive steady state NO MATTER WHAT

(POSITIVE) VALUES THE RATE CONSTANTS MIGHT HAVE.
