# Supplementary material for: Split Histidine Kinases Enable Ultrasensitivity and Bistability in Two-Component Signaling Networks
Source: PLoS Comput Biol. 2013 Mar 7;9(3):e1002949. doi: 10.1371/journal.pcbi.1002949 (PMC3591291; doi:10.1371/journal.pcbi.1002949)
Supplement: Text S5 — Results of the analytical analysis of a model with a bifunctional, non-split kinase. The file contains the reaction system considered and the report produced with the Chemical Network Tool v2.2 (http://www.chbmeng.ohio-state.edu/~feinberg/crntwin/). (DOC) [file pcbi.1002949.s017.doc]

=====================

BASIC REPORT

=====================

Reaction network:

¯¯¯¯¯¯¯¯¯¯¯¯¯¯¯¯

Ap + Y <-> A + Yp

A + Yp <-> AYp

AYp -> A + Y

Yp -> Y

A <-> Ap

Remark: None.

¯¯¯¯¯¯

Graphical Properties

====================

Number of complexes = 8

Number of linkage classes = 3:

Linkage class no. 1: {Ap + Y, A + Yp, AYp, A + Y}

Linkage class no. 2: {Yp, Y}

Linkage class no. 3: {A, Ap}

Number of TERMINAL strong linkage classes = 3:

Strong linkage class no. 1: {A, Ap}

Strong linkage class no. 2: {Y}

Strong linkage class no. 3: {A + Y}

Number of NON-TERMINAL strong linkage classes = 2:

Strong linkage class no. 4: {Ap + Y, A + Yp, AYp}

Strong linkage class no. 5: {Yp}

The network is neither reversible nor weakly reversible.

Rank Information

================

Rank of entire network = 3

Deficiency Information

======================

Deficiency of entire network = 2

Deficiency of linkage class no. 1 = 0

Deficiency of linkage class no. 2 = 0

Deficiency of linkage class no. 3 = 0

Analysis

========

This is a deficiency two network. It is an excellent candidate for application

of HIGHER DEFICIENCY THEORY (tailored mostly to networks with deficien-

cies greater than one).

Whether results will be obtained, will depend on whether or not the reaction

network has certain additional structural attributes that help reduce the problem

to a study of systems of linear inequalities.

If a network is "good", higher deficiency theory will determine, either

affirmatively or negatively, whether there are positive rate constant values

such that the corresponding mass action differential equations admit multiple

(positive) steady states. If the answer is affirmative, higher deficiency

theory will generate a sample set of rate constants and a pair of distinct

steady states that are consistent with those rate constants.

If a network is "bad", some additional nonlinear analysis might be required,

and the program might not be able to ascertain the network's capacity for

multiple positive steady states. If definite conclusions can be reached they

they will be reported. Otherwise the program will tell you that it cannot reach

a conclusion.

Higher deficiency theory will also determine, either affirmatively or

negatively, whether there can exist a set of rate constants such that the

corresponding mass action differential equations admit a positive steady

state having a zero eigenvalue (corresponding to an eigenvector in the

stoichiometric subspace). When the answer is affirmative, the theory will

produce such a set of rate constants, a positive steady state, and an

eigenvector (in the stoichiometric subspace) corresponding to an eigenvalue

of zero. Results of this kind are contained after running the Zero Eigenvalue

Report.

HIGHER DEFICIENCY REPORT: NoName1

=================================

Analysis

========

Taken with mass action kinetics, the network CANNOT admit multiple

positive steady states or a degenerate positive steady state NO MATTER

WHAT (POSITIVE) VALUES THE RATE CONSTANTS MIGHT HAVE.
